# Spatial and temporal analysis of haemorrhagic septicaemia outbreaks in India over three decades (1987–2016)

**DOI:** 10.1038/s41598-024-56213-z

**Published:** 2024-03-21

**Authors:** Mohammed Mudassar Chanda, Bethan V. Purse, Divakar Hemadri, Sharanagouda S. Patil, Revanaiah Yogisharadhya, Awadhesh Prajapati, Sathish Bhadravati Shivachandra

**Affiliations:** 1 ICAR-National Institute of Veterinary Epidemiology and Disease Informatics (NIVEDI), Ramagondanahalli, Yelahanka, Bengaluru, 560064 Karnataka India; 2 UK Centre for Ecology and Hydrology, Benson Lane, Crowmarsh Gifford, Oxfordshire OX10 8BB UK

**Keywords:** Neglected tropical disease, Cattle, Buffalo, South Asia, Haemorrhagic septicaemia, Spatial and temporal patterns, Zonal variation, Control programme, Infectious diseases, Ecological epidemiology

## Abstract

Haemorrhagic septicaemia (HS) is an economically important disease affecting cattle and buffaloes and the livelihoods of small-holder farmers that depend upon them. The disease is caused by Gram-negative bacterium, *Pasteurella multocida*, and is considered to be endemic in many states of India with more than 25,000 outbreaks in the past three decades. Currently, there is no national policy for control of HS in India. In this study, we analysed thirty year (1987–2016) monthly data on HS outbreaks using different statistical and mathematical methods to identify spatial variability and temporal patterns (seasonality, periodicity). There was zonal variation in the trend and seasonality of HS outbreaks. Overall, South zone reported maximum proportion of the outbreaks (70.2%), followed by East zone (7.2%), Central zone (6.4%), North zone (5.6%), West zone (5.5%) and North-East zone (4.9%). Annual state level analysis indicated that the reporting of HS outbreaks started at different years independently and there was no apparent transmission between the states. The results of the current study are useful for the policy makers to design national control programme on HS in India and implement state specific strategies. Further, our study and strategies could aid in implementation of similar approaches in HS endemic tropical countries around the world.

## Introduction

The livestock sector contributes significantly to global food security, and the demand for livestock and their products is increasing. The Indian livestock sector accounts for 70% (excluding poultry) of the total livestock population of South Asia, and is known to contribute to India’s Gross Domestic Product (GDP) by around 6% over the last few decades. It is expected to grow much faster in the future^1^. This sector supports the livelihoods of over 200 million rural poor^2^. Although, the livestock sector has the potential to alleviate poverty^2^, infectious diseases of livestock pose an enormous barrier to animal welfare, increased food production and livelihood improvements. A key infectious disease in this sector is ‘Haemorrhagic Septicaemia (HS)’ in cattle and buffalo.

Haemorrhagic septicaemia is a highly contagious, fatal, and septicaemic disease, caused by *Pasteurella multocida* strains especially serotype B:2 (Asian type) among five prevalent capsular serotypes (A, B, D, E, F) and sixteen somatic serotypes (1–16), which belong to the family ‘*Pasteurellaceae*’^3–6^. Susceptible animals become infected either by inhaling or ingesting the organism. The incubation period ranges from 1 to 3 days leading to sudden death without visible clinical signs. In protracted cases, the incubation period can extend up to 5 days or more. Initially, there is a high fever followed by respiratory distress (rapid and shallow breaths), septicaemia, muco-purulent nasal discharge, restlessness, oedematous swelling of the throat/brisket region, mild muscular tremors and recumbency leading to death^7,8^. On post-mortem examination, subcutaneous edema in the mandibular/brisket regions and petechial-to-echymotic hemorrhages, congestion and/or consolidation of the lungs, fibrinous pneumonia, pleurisy and pericarditis are noted^7,8^.

In areas where HS is endemic, vaccination either with alum precipitated or oil adjuvanted killed bacteria is commonly practiced. This provides 6 to 9 months of protective immunity in susceptible animals^8–10^. The vaccine is used in different states of India and is manufactured by either government or private companies. The vaccine and vaccination service is provided free of cost to the farmers. Affected animals are treated with antibiotics and antipyretics^11^. Hygienic practices, and preventing overcrowding of animals during the monsoon season are advised to prevent the occurrence of HS cases^12^. India is classified Category A in terms of the global impact of hemorrhagic septicaemia^10^**,** meaning that the disease is endemic and of utmost economic importance to the country.

Hemorrhagic septicaemia is an economically important disease of cattle and buffalo. Buffaloes are highly susceptible to HS with high morbidity and mortality in comparison to other susceptible species^13^. In another study, it was found that the morbidity rate was highest in crossbred cattle, followed by buffaloes and indigenous cattle, but the mortality rate and proportion of cases was highest in buffaloes^14^. The economic impact of HS in Cambodia was found to be $375 per animal, with benefit of $951.58 per household when vaccinated bi-annually^15^. In one study, it was found that economic losses due to morbidity were 23% and losses due to mortality were 77%^16^. Similarly, the economic losses due to HS were estimated in the state of Karnataka, India, through a primary survey of HS affected farms^17^. The loss estimates were shown to be $275, $284, and $415 per head of affected indigenous cattle, water buffaloes and crossbred cattle, respectively, with a total loss of $23.89 million/year under high incidence scenario was for the state of Karnataka^17^. However, in another study using outbreak data from 2007–2011, the loss due to HS was estimated to be ₹6816 (~ $80) per head of cattle and ₹10,901 (~ $130) per head of affected buffalo. When scaled up to the national level, the projected loss was ₹5255 crores (~ $ 800 million)^16^. The difference in the loss estimates may be due to the type of data used. The study^17^ conducted a primary survey to estimate the loss of HS, while the other study^16^ used only outbreak data which may account for the difference in estimates.

Foot-and-Mouth Disease (FMD) is the number one disease of cattle and buffalo in India, which is known to cause high economic loss to farming^18^. India has 56.7% of the worlds buffalo population and buffalo is a major dairy animal that contributes more than 50% to the country's milk production^19^. Buffalo is less susceptible to FMD and the reported mortality is lower than that of crossbred cattle. However, the economic loss due to HS is higher than FMD^16,17^. Therefore, economically, HS is a more important disease of buffalo than FMD and requires higher resource allocation to control and prevent the disease more effectively. The resource allocation can be focused on increasing testing rates and improving vaccination coverage in buffalo so that the disease can be effectively prevented.

Seasonality may or may not be present in the temporal outbreak data. If it is present, it may be due to climatic factors or temporal autocorrelation in the data as well as nature of the disease being studied. It is expected that HS outbreaks will exhibit seasonality, either due to the influence of weather or the presence of carrier/recovered animals that can act as a source of infection to other animals. This is further compounded by the waxing and waning of immunity, which lasts for 6–9 months^9,10^. The presence of carrier animals makes other animals susceptible to the disease, leading to seasonality in occurrence of the disease. Additionally, the general practice of vaccinating for HS before the onset of monsoon contributes to the seasonality in the occurrence of the disease. In animal disease outbreaks, the data tends to show spatial autocorrelation, meaning that observations that are closer to each other are more related than those farther apart. Spatial autocorrelation may occur due to the spread of the disease to neighbouring districts (for example due to the movement of animals) or due to the presence of covariates with spatial structure. HS is known to be a seasonal disease and rainfall has been associated with it’s occurrence. However, very few studies^13,20–22^ have investigated the spatial variability, seasonal pattern and periodicity in the occurrence of HS outbreaks in India. Epidemiological analysis of HS data is crucial in formulating a systematic control strategy in endemic countries^10^ by identifying seasonal patterns and spatial variability.

In India, there is no active surveillance for HS and diseases are reported by field veterinarians with the village as an “epi-unit” through a passive surveillance mechanism. Field veterinarians are assigned with many responsibilities such as treatment, vaccination, deworming and other departmental extension activities. There is always a chance of under-reporting of HS in India. The field veterinarians are under pressure to report a disease and this will affect the overall passive surveillance data used in the country. Overall, the country has weaker veterinary infrastructure compared to developed countries. There are only 65,815 veterinary centers catering to the needs of more than 500 million livestock and 800 million poultry population of the country^23^. Additionally, there are limited numbers of district and state-level laboratories to test suspected samples. However, diseases like HS with very prominent clinical signs and a high case fatality rate will seldom be missed by the farmers and are invariably brought to the notice of field veterinarians. The HS outbreak data used in the current study is passive surveillance data collected from different states of India and maintained at ICAR-NIVEDI. ‘Village’ is a spatial unit and ‘month’ is a temporal unit in the collection of HS data. The outbreaks of HS are reported by the field veterinarians and they are either clinically confirmed or laboratory confirmed cases.

Present study aimed to address following questions through the analyses of three decades (1987–2016) of HS outbreaks data from India.

1. Which zone of India is worst and most frequently affected by HS over the study period? (Spatial analysis)
2. Is seasonality of HS consistent over the study period and between regions and could this knowledge be helpful for planning control? (Singular spectrum analysis)
3. Can we detect evidence of long term inter-annual cycles in outbreak numbers? (Wavelet analysis)
4. Is there evidence of an increase or decrease in numbers of HS outbreaks over the study period, how does this trend vary between regions and can it be linked to changes in livestock numbers, control or surveillance policy? (Piecewise regression analysis)
5. What is the evidence that patterns in HS are linked or synchronous between states, due to the influence of inter-state risk factors? (Bayesian spatio-temporal model)

Overall, the analyses of HS disease data were aimed at understanding the spatial and temporal patterns of the disease as well as to provide basis for policy makers to design effective control strategy for the disease in India.

## Results

**Figure 1:**
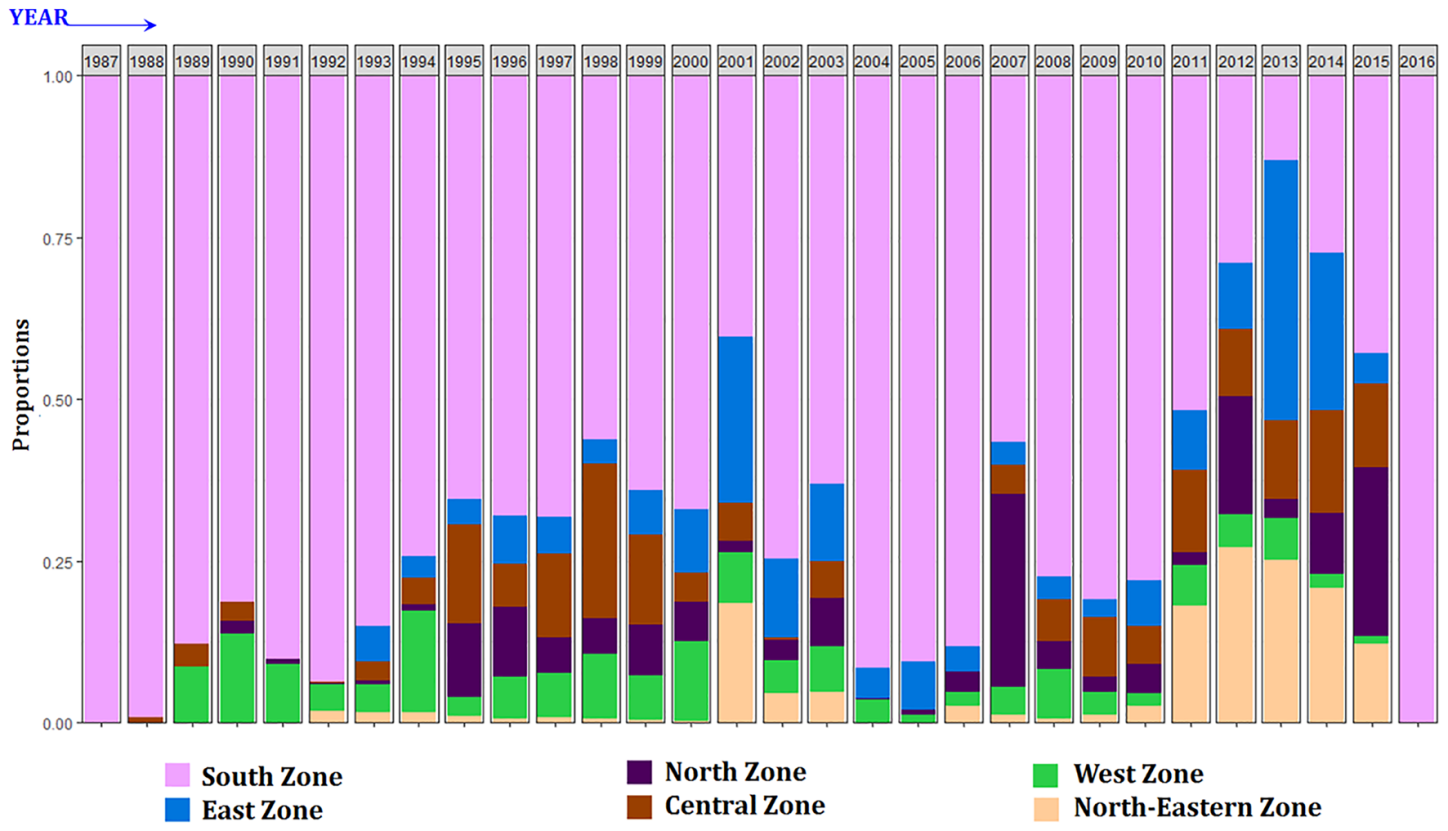
Year-wise proportion of HS outbreaks in each zone. Proportion is shown on a scale of 0–1.

**Figure 2:**
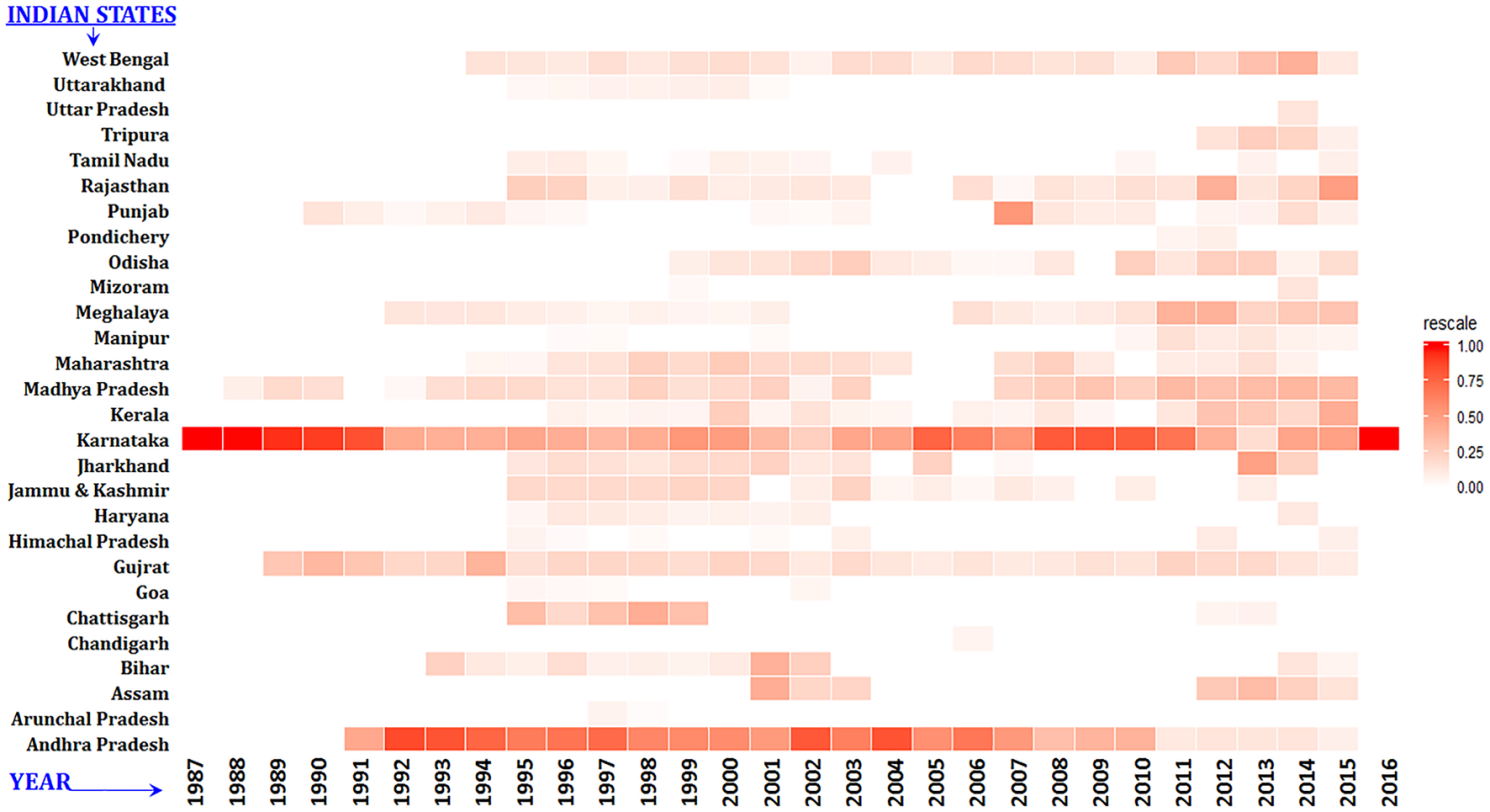
Heat map showing the state-wise distribution of HS outbreaks in different years. Proportion is shown on a scale of 0–1.

A total of 26,305 HS outbreaks were reported between January 1987 and March 2016 in India. The proportion of outbreaks in each year that were recorded in each zone was plotted to understand which zones consistently contributed to high or low number of HS outbreaks. The proportion of each year’s outbreaks that were reported in each zone is shown in Fig. 1. The South zone reported proportionately more outbreaks compared to other zones across all the years except for 2013 wherein the East zone reported more outbreaks. Karnataka and Andhra Pradesh states consistently reported the highest proportion of annual HS outbreaks across the study period (Fig. 2), with Karnataka reporting the highest proportion of outbreaks between 1987 and 1991 and again in the second half of the 2000s, and Andhra Pradesh reporting the highest proportion of outbreaks in the intervening period (1992–2005). Andhra Pradesh reported the greatest number of outbreaks (n = 10,285) followed by Karnataka with reports of 7399 outbreaks during the study period. Madhya Pradesh, Gujarat, and Punjab also frequently reported a reasonable proportion (~ 10%) of annual HS outbreaks across the study period. The remaining states reported small numbers of annual HS outbreaks sporadically. Overall, the South zone reported the maximum proportion of the outbreaks (70.2%), followed by the East zone (7.2%), the Central zone (6.4%), the North zone (5.6%), the West zone (5.5%) and the North-East zone (4.9%). The Central zone has a higher proportion of cattle (26.71%) and buffalo (37.89%) compared to other zones. The North zone has 11.84% and 23.78%, the East zone has 25.72% and 9.27%, the South zone has 15.36% and 13.78% and the North-East zone has 6.96% and 0.53% proportion of cattle and buffalo population respectively.

Proportion of HS outbreaks in different states in each zone is shown in Fig. S1A–F. Proportion of HS outbreaks in comparison to buffalo and cattle population in each zone is given in Fig. S2. State-wise proportion plot of cattle and buffalo population in each state (Fig. S3) and state wise population maps of cattle and buffalo are shown in Fig. S4.

### Autocorrelation function (ACF), partial autocorrelation function (PACF) and decomposition of time series

Autocorrelation functions (ACF) and partial autocorrelation functions (PACF) plots of different zones are shown in Figs. S5A–F and S6A–F. All the zones exhibit non-stationarity which was further confirmed with Augmented Dickey-Fuller (ADF) test (Table S1). The trend of different zones is shown in Fig. S7A–F.

### Decomposition of HS outbreaks using Singular Spectrum Analysis (SSA) at zonal level

**Figure 3:**
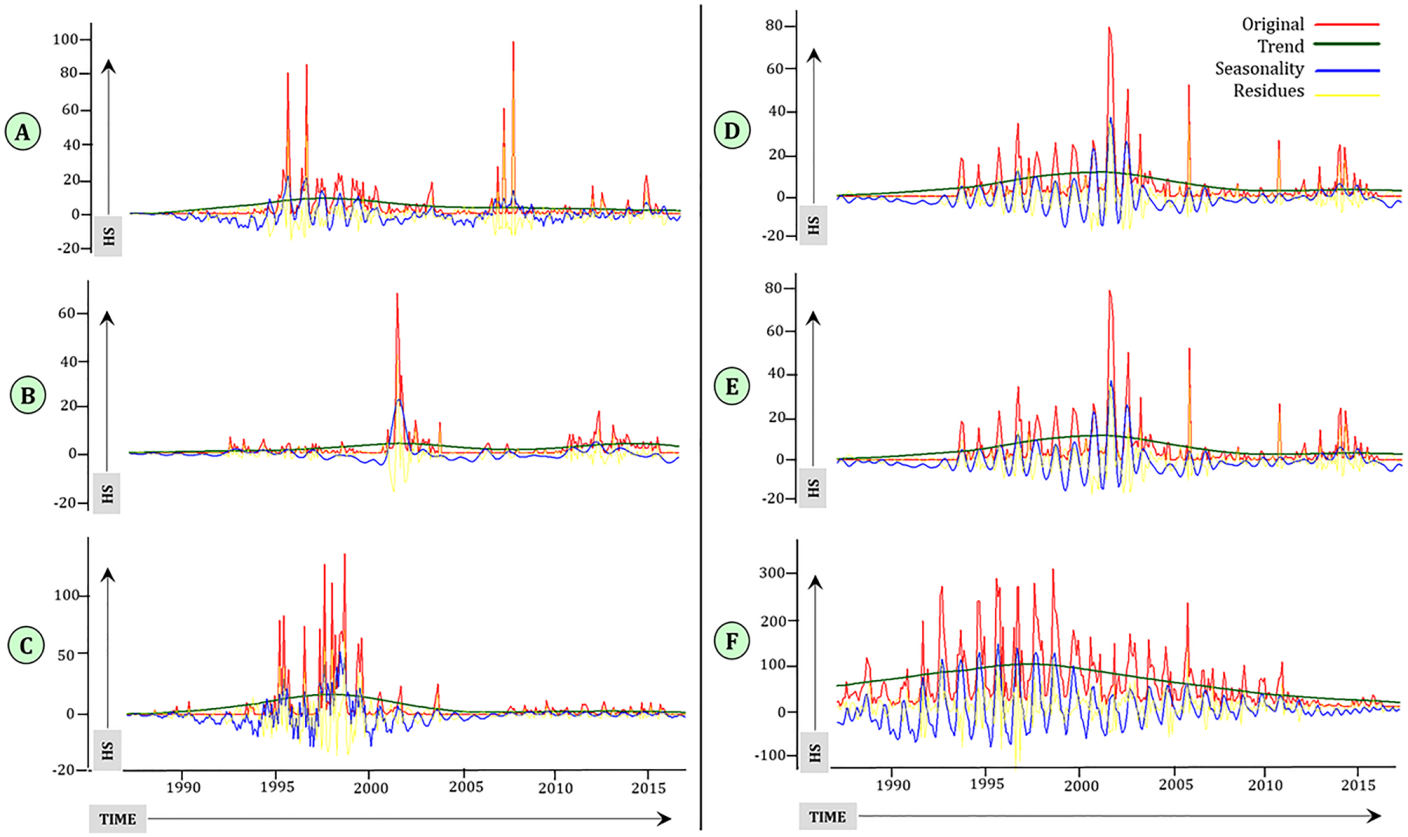
Decomposition of the monthly HS time series using singular spectrum analysis. The original time series is overlaid with the seasonality, trend and residual components.

The North zone showed an irregular pattern with peaks around 1995–2000 and 2006–2008.There is a seasonal pattern during the initial part of the series, and there was residual variability (Fig. 3A). The North-East zone time series shows one peak around the year 2001–2002.There was residual variation that coincided with the peaks in HS outbreaks and was not explained by seasonality or the trend (Fig. 3B). There was also presence of the seasonality in the time series and residual variations that coincided with the peaks of HS outbreaks in the Central zone (Fig. 3C). The East zone time series shows a trend, seasonality and residual variation, indicating that there were regular reports of HS outbreaks since the year 1994 and gradually started decreasing from the year 2005 onwards (Fig. 3D). There was the presence of seasonality coupled with residual variation in the Eastern zone. There was also presence of the seasonality and residual variation in the time series of the West zone (Fig. 3E). There was seasonality in HS outbreaks and residual variation after accounting for trend and seasonality in the South zone (Fig. 3F).

### Wavelet transformation of HS time series at zonal level

**Figure 4:**
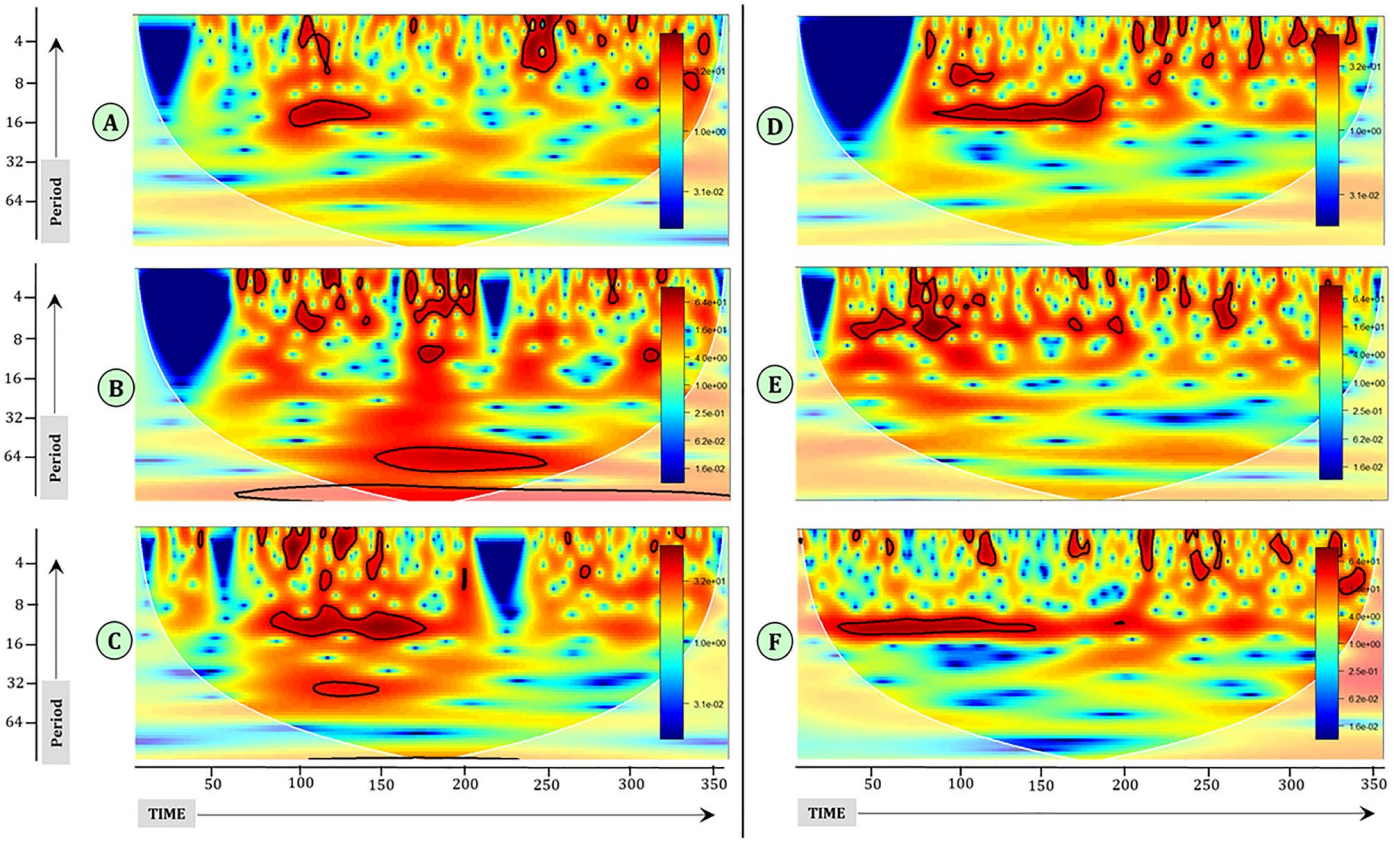
Wavelet transformation to detect dominant frequencies in monthly HS outbreaks. The white dotted line is the cone of influence indicating the region of time and frequency where the results are not influenced by the edges in the data and are reliable. The solid black line corresponds to the 95% confidence interval and the areas within this black solid line indicate significant variability at the corresponding periods and times. X-axis: time in months from January 1987 (= month 1), Y-axis: localised periodicity in months.

Wavelet transformation indicated that there was seasonality (around 12 months), but an absence of significant long-term periodicity in the North zone (Fig. 4A). The wavelet transformation detected dominant, significant periodicity around 5–6 years and also around eight years for the North-East zone (Fig. 4B). The transformation reflected seasonality around 12 months during the initial phase of the time series and also at around 3 years, but again for a shorter time for the Central zone (Fig. 4C). It reflected the seasonality of around 12 months during the initial phase of the series (Fig. 4D) for East zone. It shows that there was seasonality but not consistent along the time series for West zone (Fig. 4E). The wavelet transformation reflected that there was significant seasonality around 12 months for the first half of the time series for South zone (Fig. 4F).

### Piecewise regression analysis to identify break points in years and months

**Figure 5:**
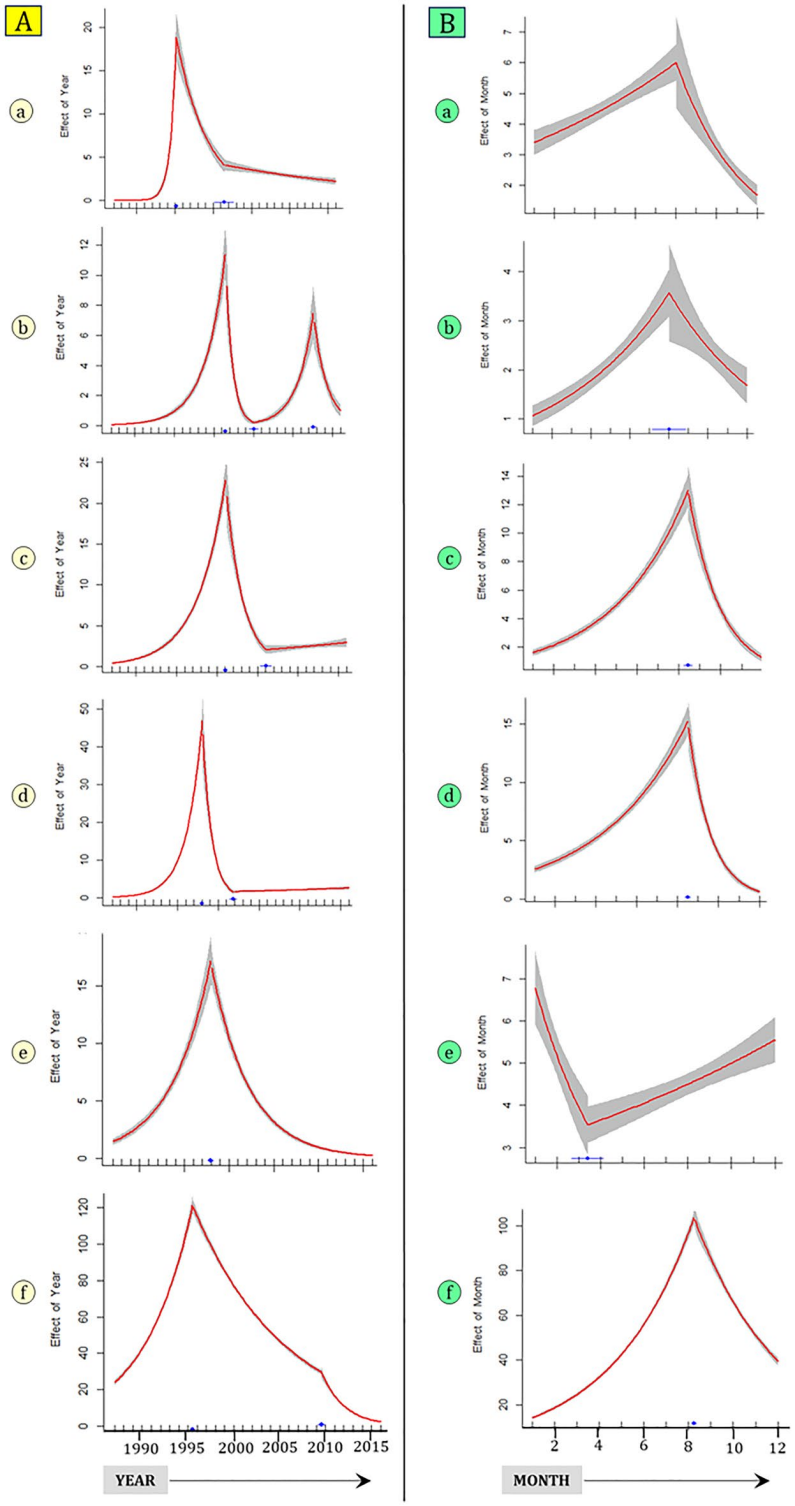
(**A**) Plots of piecewise regression analysis with year as dependent variable. Grey filled areas are 95% confidence interval. Blue is the break point identified. (**B**) Plots of piecewise regression analysis with month as dependent variable. Grey filled areas are 95% confidence interval. Blue is the break point identified. (a) North zone, (b) North East zone, (c) East zone, (d) Central zone, (e) West zone and (f) South zone.

From the year 1995 there was a decreasing trend which further decreased from 2001 onwards [Fig. 5A(a)] in the North zone. In the North-East zone, in the year 2000, there was an increase in the number of outbreaks, which decreased in the year 2008 and there was a further increase in the year 2011 [Fig. 5A(b)]. There was a peak of HS outbreaks in the year 2001 and a decreasing trend from the year 2005 as detected in the East zone [Fig. 5A(c)]. In the Central zone, there were many outbreaks during the years 1995–2000 and year 1998 and 2000 were detected in break point analysis. In the year 1998, there was an increasing trend and decreasing trend from the year 2000 for East zone [Fig. 5A(d)]. In West zone, there was increasing trend from the year 1989 and a decreasing trend from the year 1997 as detected in the break point analysis [Fig. 5A(e)]. The South zone time series indicated that the HS outbreaks had been reported regularly since the year 1987, and there is a decreasing trend from the year 1995 and a further decreasing trend from the year 2009 as detected in the break point analysis [Fig. 5A(f)].

Piecewise regression analysis to identify break points with Year and Month as independent variables for each zone is shown in Table 1. Except for the West zone, all the other zones identified the eighth month (August) as a change point with outbreaks decreasing after that [Fig. 5B(a–f)].

**Table 1:** Change point detection using piece wise regression analysis using year and month as independent variable.

Zone	Change point years ± SE	Change point month ± SE
North zone	1995 ± 0.06	8.000 ± 0.344
	2001 ± 0.61	
North-East zone	2001 ± 0.18	8.039 ± 0.429
	2008 ± 0.31	
	2011 ± 0.13	
Central zone	1998 ± 0.09	8.492 ± 0.075
	2001 ± 0.23	
East zone	2001 ± 0.155	8.462 ± 0.093
	2005 ± 0.368	
West zone	1989 ± 0.86	3.387 ± 0.364
	1998 ± 0.19	
South zone	1995 ± 0.09	8.244 ± 0.053
	2009 ± 0.19	

The change point is shown with ± standard error (SE).

### District wise spatial analysis of HS outbreaks in all the zones

**Figure 6:**
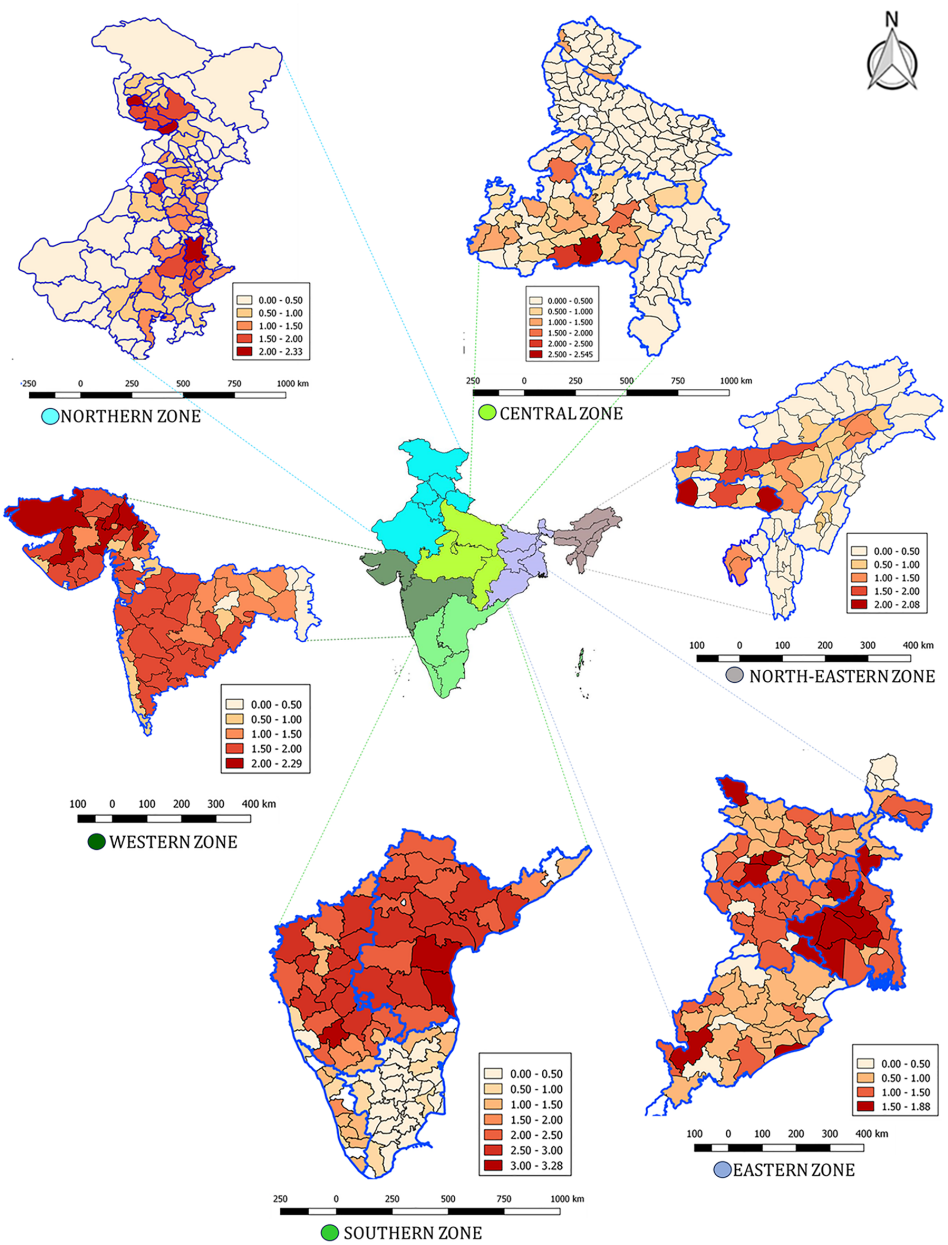
Cumulative (1987–2016) spatial distribution of HS outbreaks in the zone (log_10_ transformed) in different zones of India.

The state-wise spatial distribution of outbreaks in all the zones is shown in Fig. 6. For the North zone, it indicates that there were few outbreaks reported from Uttaranchal, the North and North-Western parts of Rajasthan and districts of other states like Haryana and Punjab reporting outbreaks. In the North-East zone, HS outbreaks had not been reported from the majority of the districts of Arunachal Pradesh, Nagaland and Mizoram. However, the majority of the districts from Assam and Meghalaya had reported HS outbreaks. In the Central zone, outbreaks of HS had been reported from Madhya Pradesh and other states like Uttaranchal, Uttar Pradesh, while Chhattisgarh had reported sporadic outbreaks. In the East zone, HS outbreaks had been reported from all the states with the maximum reports from districts of West Bengal. The majority of the districts of Gujarat and Maharashtra had reported HS outbreaks in Western zone. Majority of the districts of Karnataka and Andhra Pradesh had reported HS outbreaks followed by Kerala. Tamil Nadu reported sporadic HS outbreaks.

### Spatial model

The spatial models were fitted to different zones separately, and the results are shown in Table 2. The comparison of models based on the lowest DIC shows that *iid* models are best for all the zones indicating the presence of distinct district-level factors contributing to the district- level variability in the occurrence of HS outbreaks. The spatial unstructured heterogeneity maps are shown in Fig. S8A–F.

**Table 2:** Spatial and spatio-temporal models.

A. Spatial models			
Zone	*BYM*	*Besag*	*iid*
South zone	657.24	663.98	657.22
Central zone	–	–	339.74
Eastern zone	673.24	678.88	673.23
North-East zone	298.45	308.90	298.42
North zone	497.53	507.42	497.23
West zone	428.61	440.48	428.72

B. Space–time models			
Model	DIC		
Model without spatial and temporal autocorrelation	235,817.60		
Model with spatial and temporal autocorrelation	22,803.39		
AR (1) + *besag*	22,804.68		
AR (1) + *iid*	22,803.58		
Model with spatial and temporal autocorrelation and Type-1 interaction	2743.68		

(A) DIC of different spatial models (*BYM*, *Besag* and *iid*) fitted for different zones. It shows that the iid models are the best models for all zones based on the DIC values.Note: For Central zone, DIC could not be computed for *Besag* and BYM models and it was computed only for iid model. (B) Comparison of different spatio-temporal models with DIC. Model with spatial and temporal autocorrelation and Type-1 interaction was the best model with lowest DIC.

### Annual variability in reported HS outbreaks at State level

**Figure 7:**
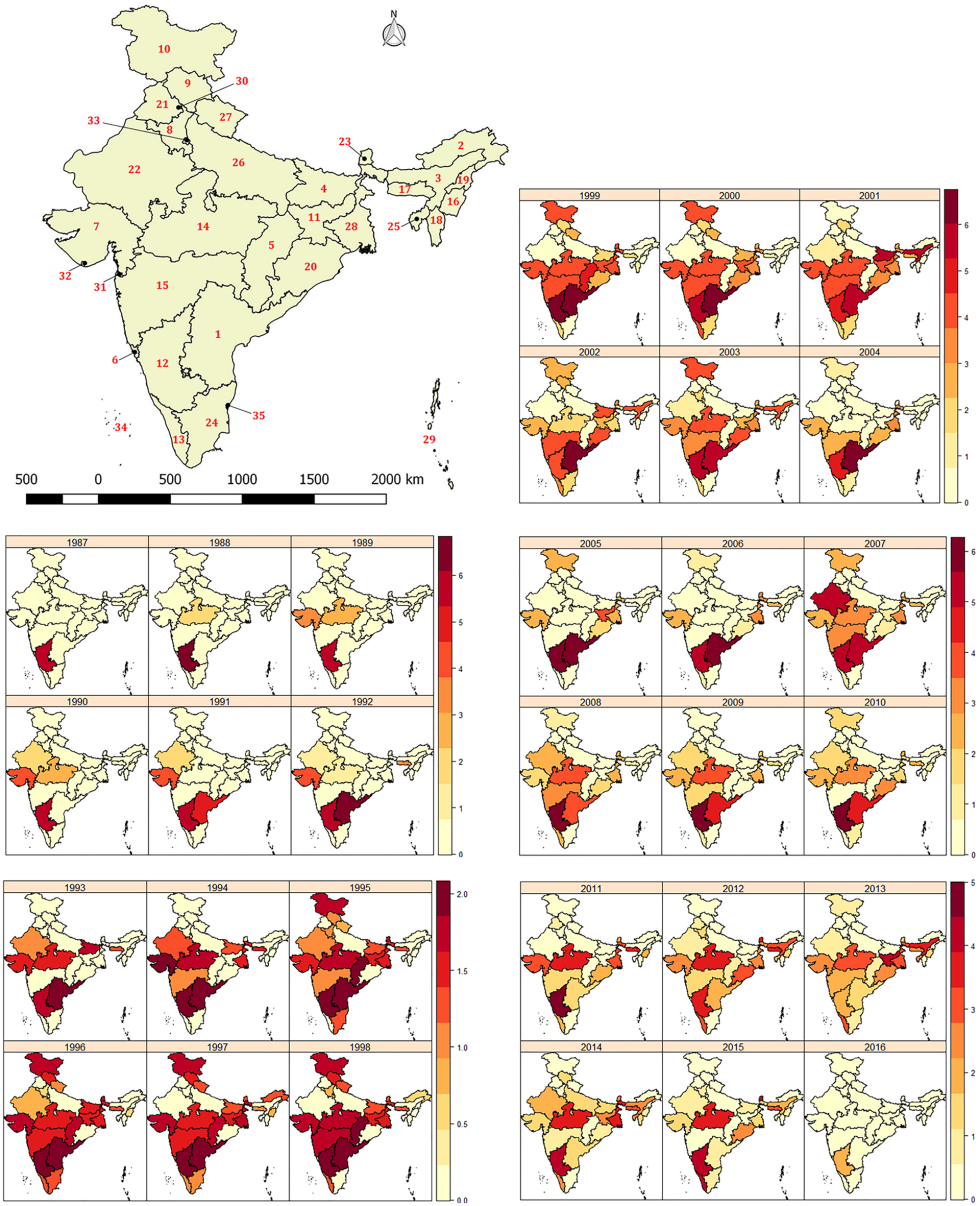
Spatio-temporal patterns of HS outbreaks in India (1987–2016). The HS outbreak data were log_10_ transformed. Number denotes names of different states and they are: 1, Andhra Pradesh; 2, Arunachal Pradesh; 3, Assam; 4, Bihar; 5, Chhattisgarh; 6,Goa; 7, Gujarat; 8, Haryana; 9, Himachal Pradesh ; 10, Jammu and Kashmir; 11, Jharkhand; 12, Karnataka; 13, Kerala; 14, Madhya Pradesh; 15, Maharashtra; 16, Manipur; 17, Meghalaya; 18, Mizoram; 19, Nagaland; 20, Odisha; 21, Punjab; 22, Rajasthan; 23, Sikkim; 24, Tamil Nadu; 25, Tripura; 26, Uttar Pradesh; 27, Uttaranchal; 28, West Bengal; 29, Andaman and Nicobar; 30, Chandigarh; 31, Dadra and Nagar Haveli; 32, Daman and Diu; 33, Delhi; 34, Lakshadweep; 35, Pondicherry.

The severity of the outbreaks in each state and each year varied (Fig. 7). The NADRES (National Animal Disease Referral Expert System) database of ICAR-NIVEDI started collating outbreak data since the year 1987. In 1987, Karnataka state reported the maximum number of outbreaks (n = 243), and subsequently, other states started reporting. During the years 1995–2003, most of the states reported HS outbreaks (except Nagaland, Andaman and Nicobar Islands, Lakshadweep, Dadar, Daman, Delhi). Thereafter, there was a decrease in the number of outbreaks reported.

Spatio-temporal patterns in annual outbreak numbers at the state-level are best explained by a model that combines spatially unstructured (*iid*), spatially structured (*Besag*) and temporal components (AR (1)), as well as a type-1 interaction (Table 2B).

## Discussion

Understanding the spatial and temporal patterns of animal diseases is critical in planning systematic surveillance in at-risk areas during high-risk periods so that effective prevention and control strategies can be formulated. In the present study, thirty-year HS outbreak data was analysed to identify the spatial and temporal patterns of HS outbreaks in different zones of India using spatial and temporal methods. We used a spatio-temporal model to identify the inter-annual variability of the disease in different states of India. There is seasonality in the occurrence of HS outbreaks in India with the peak of outbreaks happening in August. No inter-annual periodicity was detected in all zones except the North-East zone, indicating that long term immunity or climate extremes may not be playing a role in determining the occurrence of HS outbreaks. There are some district-level factors that determine the spatial variability of HS outbreaks in different zones of India, and there is no apparent transmission between states. Hence, this study shows that there is a need for state specific or zone specific policies to prevent and control HS in India.

There will be either short-term (seasonal) or long-term (inter-annual) dependencies in the temporal data and hence appropriate methods should be used to quantify their importance. Seasonality was detected across all zones except, for the North-East zone where seasonality was not detected. There was no significant periodicity detected in wavelet analysis for all the zones, except for the North-East zone. In the North-East zone, dominant and consistent periodicity was detected around 5–6 years and around 8–10 years (Fig. 4B). The dominant periodicity around 5–6 years, indicates that HS in this region may occur with a periodicity of 5–6 years.

Piecewise regression analysis detected specific years and months in which there was a trend (increasing or decreasing). There was non-linearity in the occurrence of the outbreaks at the yearly and monthly levels. The varying trend in different years indicated specific changes in policy in certain years, such as vaccination coverage, changes in livestock population (especially cattle and buffalo), or the influence of rainfall which need to be further investigated. The varying monthly and yearly trend detection using piecewise regression is advantageous over other linear models which fail to capture the non-linearity in the occurrence of HS outbreaks in India. The varying trend indicates that the disease can be prevented in the future by adopting a specific vaccination schedule for each zone or state to protect the animals against HS.

Spatial analysis of diseases can help in the eradication of infectious diseases by implementing cost-effective disease management schemes in newly predicted areas with spatial structure. In this study, we employed different spatial models for each zone to detect spatial patterns in the disease. The *iid* models outperformed the *Besag* or *BYM* models for all the zones (Table 2A). This indicates that there were certain district level factors (hosts, weather, land cover etc.,) that were different in each district, and those factors were more important rather than factors that are spatially autocorrelated (Fig. S8). In addition, certain states contributed to more outbreaks (Fig. S1A–F) to that zone compared to other states, and hence, this could influence the spatial pattern in outbreaks. Within the endemic zone or state, there were some districts that were affected more severely than others. Recent studies on whole genome analysis and genetic typing revealed that there is genetic diversity of Indian isolates of *P. multocida* within^24^ and between the geographical regions^25^. The factors responsible for variability in HS outbreaks at the district level can be further investigated by incorporating covariates. There are no reports on spatial analysis of HS data from India using statistical methods except a recent study that showed seroprevalence of HS was significantly higher in indigenous cattle compared to crossbred cattle^26^, but these methods are commonly employed to analyse livestock diseases in other countries. The spatial pattern of Foot-and-Mouth disease (FMD) in China was analysed to detect spatial autocorrelation and hotspots for the disease^27^. A continental risk map for the occurrence of Rift Valley fever was prepared using a Bayesian spatial model by including environmental covariates^28^. A Bayesian model was used to identify the risk factors for the occurrence of Classical Swine Fever (CSF) in Bulgaria^29^. Exploratory spatial data analysis and the Empirical Bayes method were used to identify high-risk areas for human brucellosis which can be useful in allocating resources to reduce the burden of the disease^30^.

Spatial and temporal patterns of animal diseases are important not only to contain the diseases within a country but can also help to prevent spread to neighbouring countries^31^. Space–time clusters were detected for bluetongue outbreaks in the Balkan Peninsula using scan statistics^32^. Bayesian survival models were used to understand the spatio-temporal dynamics of pneumonia in bighorn sheep^33^. Spatio-temporal analysis of anthrax outbreaks in Ukraine during the past century (1913–2012) identified disease hotspots and found that outbreaks have reduced during the latter part of the century^34^. In the present study, space–time multi panel plots showed that the HS outbreaks had inter-annual variability in each state (Fig. 7). Inter-annual variability may be either due to variability in weather or because of an effective vaccination strategy in certain years compared to other years. Space–time data can be analysed by different methods^35^. In this study, space–time data on HS outbreaks in each state was analysed using a Bayesian Poisson Generalised linear model accounting for spatial and temporal autocorrelation. The model with spatial and temporal autocorrelation random effects outperformed the models without random effects indicating the importance of these terms in explaining the state-wise inter-annual variability of HS outbreaks (Table 2B). Interestingly, *iid* model for spatial terms and AR (1) for the temporal component was better than the model with *Besag* component and AR (1). Additionally, the inclusion of type-1 interaction (interaction between *iid* of spatial component and *iid* of temporal component) further reduced the model DIC compared to the model without the interaction term (Table 2B). This indicates that there were some state-specific policy changes in certain years (e.g. effective vaccination) or change in state-specific covariates (weather, host, or land cover) that led to the inter-annual variabilities within and between the states. The model presented in this study can be extended to include covariates either at the district or state level for developing an early warning system for HS in India.

There is a need to practice vaccination at least 3 months before^10^ to achieve maximum protection. Each zone was different in seasonality of the disease and hence, specific months should be selected to achieve maximum protection. There was also spatial variability in the occurrence of HS outbreaks between and within zones. Hence, resources (vaccines, personnel etc.) could be allocated accordingly to reduce the economic burden of the disease in India. The comprehensive epidemiological data analyses of HS outbreaks presented in this study are likely to assist policy-makers in systematic planning of control strategy for the disease in India.

On the basis of analyses presented in this study, it is evident that despite control strategies adopted by respective state animal husbandry departments for annual HS vaccination (either alum precipitated or oil adjuvant-based killed HS vaccine) in selected endemic regions over several years, HS outbreaks were recorded which might be due to improper vaccination strategy (month of vaccination, areas for vaccination, etc.,). Although the average loss per animal due to mortality following HS was estimated to be around $275 (indigenous cattle), $284 (water buffaloes) and $415 (crossbred cattle) in different animals, Benefit-Cost Analysis (BCA) of the HS vaccination was estimated at different incidence scenarios such as 5.97:1 (high), 4.48:1 (medium) and 2.98:1 (low)^17^, but, the countrywide actual impact of morbidity/mortality due to HS alone or in association with concurrent infections/varying risk factors at different agro-climatic regions needs to be estimated. Haemorrhagic septicaemia continues to haunt the health status (although at a decreasing trend) of the Indian livestock population, a pragmatic National Control Programme on HS is imminent^10,36^, in order to reduce the disease burden, which was tentatively scaled up to cost approximately ₹5255 crore per annum (~ $ 800 million)^16^. Furthermore, a serious revisit to the existing strategy involving widely used vaccine strain, vaccination schedule, adjuvant and delivery systems should be a priority for the sustainable development of the livestock sector under the National Livestock Mission (NLM) launched by DADF (Department of Animal Husbandry, Dairy and Fisheries), Government of India^36^. There is a need to develop a prevention and control strategy for the disease by utilising the resources in high-risk areas as well as high-risk periods, and targeted surveillance in order to reduce the burden of HS as it could be curtailed by implementing a pragmatic HS National control program in India.

## Conclusion

Overall we found that the South zone is the worst and most frequently affected zone among all the zones. Therefore, resource allocation for surveillance and vaccination programs should be more concentrated in the South zone. In the other zones, there is a need to improve the surveillance for the early detection of HS cases. Seasonality was found in all the zones, with outbreaks being more common in the month of August. Therefore, vaccination can be carried out in the months of May–June for better protection to the susceptible population. Except for the North- East zone, no long-term periodicity was detected in any of the other zones. The absence of long term periodicity indicates that there is no impact of long term changes in climate on the occurrence of HS outbreaks that needs to be further investigated. Non-linearity was detected in the monthly and yearly occurrence of HS outbreaks and some break-points were identified. These breakpoints, whether occurring monthly or yearly, may be due to changes in vaccination policy or changes in livestock numbers, mainly cattle and buffalo. The spatial variation of HS outbreaks at the district level may be attributed to some distinct district level factors and there is no evidence of transmission between districts. The analyses also revealed that there is no apparent transmission of HS between states, suggesting that state-level HS policies can be formulated for effective prevention and control of HS in India.

## Materials and methods

### Data from NADRES

#### Haemorrhagic septicaemia outbreak data

The HS disease data used in the present analyses are both clinically suspected and confirmed based on laboratory methods (microscopy/bacterial isolation/PCR assays). District-level (admin-2) monthly HS outbreak data (Jan 1987–March 2016) were obtained from NADRES (National Animal Disease Referral Expert System) database of ICAR-NIVEDI (Indian Council of Agricultural Research-National Institute of Veterinary Epidemiology and Disease Informatics), which maintains the HS database for India and has collated outbreak data every month from different sources since 1987. A village is considered as an ‘epi unit’ for the reporting of HS outbreaks, and a month is considered as the time unit. The reason for considering village as an ‘epi unit’ is due to common husbandry practices within a village and surveillance reports are compiled every month.

#### Aggregation of HS outbreaks data at zonal level

First, district-level HS outbreak data were aggregated across all states to calculate the total sum of outbreaks in each month for every state. Then, the state-level monthly data was aggregated for each zone: North (Chandigarh, Delhi, Haryana, Himachal Pradesh, Jammu and Kashmir, Punjab and Rajasthan), North-East (Assam, Arunachal Pradesh, Manipur, Meghalaya, Mizoram, Nagaland, Sikkim and Tripura), East (Bihar, Jharkhand, Odisha and West Bengal), Central (Chhattisgarh, Madhya Pradesh, Uttaranchal and Uttar Pradesh), West (Dadra and Nagar Haveli, Daman and Diu, Goa, Gujarat and Maharashtra) and South zones (Andhra Pradesh, Karnataka, Kerala, Lakshadweep, Puducherry, Tamil Nadu and Telangana). Telangana, a new state carved out of Andhra Pradesh in 2011was included in the analysis using the combined data of the Andhra Pradesh data. The zonal data was used for singular spectrum analysis, wavelet analysis and zonal piece wise regression analysis.

#### Aggregation of HS outbreaks at district level

The district-level monthly HS outbreaks data was aggregated for each district to calculate the sum of outbreaks across all years resulting in district-level sum of outbreaks. This data was utilised for spatial analysis of HS outbreaks at district-level for each zone.

#### Aggregation of HS outbreaks data at state level

The monthly district-level HS outbreak data was aggregated for each state to calculate the sum of outbreaks in each year. This data was used to analyse spatio-temporal patterns in the data.

### Methods

#### Zonal level time series analysis

##### Decomposition of HS outbreaks using Singular Spectrum Analysis (SSA) at zonal level

SSA was performed on the zonal-level HS outbreaks data for all the zones. The SSA algorithm decomposes the time series into different components and each component can be identified as either a trend, periodic component, or noise. Subsequently, the original time series was reconstructed by removing the stochastic noise^37^. Singular spectrum analysis can also detect non-linearity in the time series. SSA was implemented using the R package RSSA^37^.

#### Wavelet transformation of HS time series at zonal level

Wavelet analysis has been used in epidemiological time series^38^ to identify seasonality and periodicities in the data. Wavelet transformation of the time series can be performed either by discrete (Discrete Wavelet Transform DWT) or continuous (Continuous Wavelet Transform, CWT) method^38,39^. The CWT was used in the present study for each zone. Wavelet analysis was performed on the monthly time series using the *biwavelet* package in R^40^.

#### Piecewise regression analysis

For each zone, piecewise regression models were fitted to estimate long-term trends (increasing or decreasing). Two sets of models were fitted using Year and Month as independent variables. There are two steps in fitting piece wise regression models. In the first step, a standard Poisson regression model was fitted to each zonal time series with year as the independent variable and HS outbreaks as the dependent variable. In the second step, the model was refitted using the segmented function in the *segmented* package^41^. The same steps were repeated as above with month as the independent variable. This method identifies piecewise linear relationships between year or month and HS outbreaks and provides an estimate of approximate change points/break points, for example, years or months marked by an increasing or decreasing trend.

### District level spatial analysis of HS outbreaks for different zones

#### District level spatial model description

The probability function for Y is demonstrated using

$${\text{Pr}}\left({\text{Y}}=\frac{{\text{y}}}{\upmu }\right)=\mathrm{Poisson }({\mu }_{i})$$

$$\mathrm{Log }({\mu }_{i})= {\beta }_{0}+ {\upsilon }_{i}+ {\nu }_{i}$$

$${\upsilon }_{i}/\nu _{i} \ne 1 \sim Normal ({m}_{i} , {S}_{i}^{2})$$

$${m}_{i}=\frac{\sum j\epsilon {N}_{(i)}{\upsilon }_{i}}{\ne {N}_{(i)}}$$

$${S}_{i}^{2}=\frac{{\sigma }_{\upsilon }^{2}}{\ne {N}_{(i)}}$$

where $\beta {0}$ is the intercept.$\upsilon i$ is a structured spatial component assuming Besag-York-Mollie (BYM) specification^42^, modelled using an intrinsic conditional autoregressive structure (iCAR).

$$\ne N(i)$$ is the number of districts that share boundaries with the *i*-th district, and $$\nu i$$ is the unstructured spatial effect in each district, modelled using an exchangeable prior $$\nu i$$ ~ Normal (0, $$\sigma$$$$\upsilon$$^2^). iCAR is based on a set of districts that share boundaries for which an adjacency matrix was defined, listing for each district, all other districts with which it shares a boundary or adjacency. Weights are defined for those adjacencies, and have a value of one when two districts share a boundary and a value of zero when they do not.

District level spatial models were fitted using Bayesian Poisson generalised linear model for each zone implemented in INLA-R^43^. Three different models were implemented namely *BYM*, *Besag* and *iid* model for each zone separately. Deviance Information Criteria (DIC)^44^ was used to compare different models. All the maps were prepared using the free and open source QGIS^45^**.**

### State level spatio-temporal analysis of HS outbreaks

#### Space–time multipanel plots

Exploratory analysis of spatio-temporal data is important to understand the variability in the outbreaks in different years across different states. Space–time multi panel plots were generated using the *space–time* package in R^46^.

#### Spatio-temporal model

The annual number of outbreaks across each state was fitted using space–time Bayesian Poisson Generalised linear model accounting for spatial and temporal autocorrelation to understand the spatial and temporal dynamics of HS outbreaks across states. The models were implemented in INLA-R^43^. In addition, a model was fitted with space–time type-1 interaction. In type-1 interaction the DIC^44^ was used to compare different models.

$${Y}_{it}\sim Poisson \left({E}_{it }{\mu }_{it}\right)$$

$$\mathrm{Log }({\mu }_{\mathit{it}})= {\beta }_{0}+ {\varepsilon }_{i}+ {S}_{i }+ {\gamma }_{t}+{S}_{t}$$

$$\varepsilon_{i}/\varepsilon _{j}=1\sim \mathrm{Normal }({m}_{i }{s}_{i}^{2})$$

$${m}_{i}=\frac{\sum j \epsilon N \left(i\right){\varepsilon }_{j}}{\ne N}$$

$${S}_{i}^{2}=\frac{{\sigma }_{\varepsilon }^{2}}{\ne {N}_{(i)}}$$

$${\gamma }_{t}=\sum_{j=1}^{p}{\rho }_{j}{\gamma }_{t-1}+{Z}_{t}$$

$${Z}_{t}\sim N (0, {\sigma }^{2}\gamma )$$

$${\frac{{\tau }_{t}}{{\tau }_{j}}}_{t}=1-Normal ({m}_{t}, {s}_{t}^{2})$$

$${m}_{t}=\frac{\sum j\epsilon {N}_{(t) }{\tau }_{j}}{\ne N}$$

$${s}_{t}^{2}=\frac{{\sigma }_{\tau }^{2}}{\ne {N}_{(t)}}$$

where $$\beta {0}$$ is the intercept.$$\varepsilon i$$ is the structured spatial component assuming Besag-York-Mollie (BYM) specification^42^, modelled using an intrinsic autoregressive structure (iCAR). $$\ne N(i)$$ is the number of states that share boundaries with the i-th one (i.e. its neighbouring state). $$si$$ is the unstructured spatial effect in each state modelled using an exchangeable prior, $$si$$ ~ Normal (0, $$\sigma$$$$\varepsilon$$^2^). $$\gamma t$$ is the structured temporal component assuming (i) an AR (1) structure yy^47^; (ii) stationarity of the data over time and (iii) all the observations are regularly spaced in time. $$\tau t \,$$ is the unstructured temporal heterogeneity in each district modelled using $$st\sim N(0,\sigma^{2} \gamma )$$. y_it_ denotes the number of HS outbreaks occurring in year t t(t = 1,….T) in each state i (i = 1,….I). It is assumed that the number of outbreaks y_it_ for each state i, in year t, has a Poisson distribution with parameters µ_it_ and probability π_it_ with a log link, where linear predictor µ_it_ decomposes additively into time and space dependent effects. Space–time interaction term was added to the model to fit type-1 interaction model.

### Supplementary Information

Supplementary Information.

## Data Availability

The Haemorrhagic septicaemia outbreaks data are provided in materials and methods in the form of time series plots, proportion plots and spatial distribution maps. Prior permission from Director, ICAR-NIVEDI is required for utilising the data for any purpose.
