# Supplementary material for: Spatial and temporal analysis of haemorrhagic septicaemia outbreaks in India over three decades (1987–2016)
Source: Sci Rep. 2024 Mar 21;14:6773. doi: 10.1038/s41598-024-56213-z (PMC10957987; doi:10.1038/s41598-024-56213-z)
Supplement: Supplementary file 1 — Supplementary Information. [file 41598_2024_56213_MOESM1_ESM.docx]

**Supplementary information**

**Spatial and Temporal Analysis of Haemorrhagic Septicaemia Outbreaks in India over Three Decades (1987-2016)**

Mohammed Mudassar Chanda^1🖂^, Bethan V. Purse^2^, Divakar Hemadri^1^, Sharangouda S. Patil^1^, Revanaiah Yogisharadhya^1^, Awadhesh Prajapati^1^, and Sathish Bhadravati Shivachandra^1^

^1^ICAR-National Institute of Veterinary Epidemiology and Disease Informatics (NIVEDI), Ramagondanahalli, Yelahanka, Bengaluru- 560064, Karnataka, India

Phone: 0091-80-23093100 (Ext: 141)

Fax: 0091-80-23093222

E-mail: ^🖂^Mudassar.Mohd@icar.gov.in and [chandamudassar@gmail.com](mailto:chandamudassar@gmail.com)

^2^UK Centre for Ecology and Hydrology, Benson Lane, Crowmarsh Gifford, Oxfordshire, OX10 8BB, United Kingdom (UK).

**S1. Zone and state level variability in numbers of HS outbreaks**

# **Intra zone wise proportion maps:** Year-wise proportion of outbreaks in each zone was plotted to know the distribution of HS outbreaks in different states within a zone (Fig. S1 A-F).

*Figure S1: Year-wise proportion of HS outbreaks and contribution of each state in different zones. (A) North zone, (B) North East zone, (C) Central zone, (D) East zone, (E) West zone, (F) South zone.*


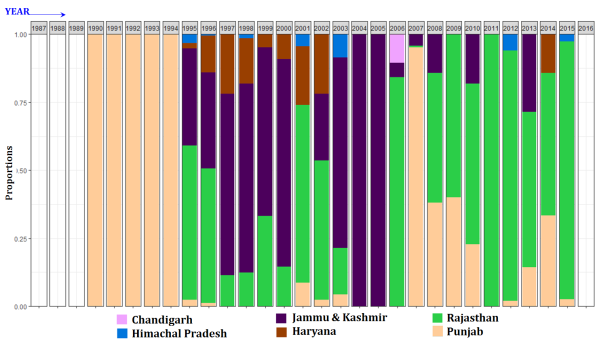


**A**

In North zone, Punjab contributed to more number of outbreaks from 1990-1994 and in year 2007. In year 1995 and 1996, Rajasthan contributed more number of outbreaks. From year 1997 to 2000, Jammu & Kashmir contributed more than other states. Rajasthan contributed more number of outbreaks from year 2008 until 2015. Within the North zone, Rajasthan (36.88%), Punjab (29.5%) and Jammu & Kashmir (27.33%) proportionately contributed more outbreaks compared to other states (Fig. S1A). Rajasthan has an equal proportion of cattle and buffalo population (Cattle: 50.66%, Buffalo: 49.33%).


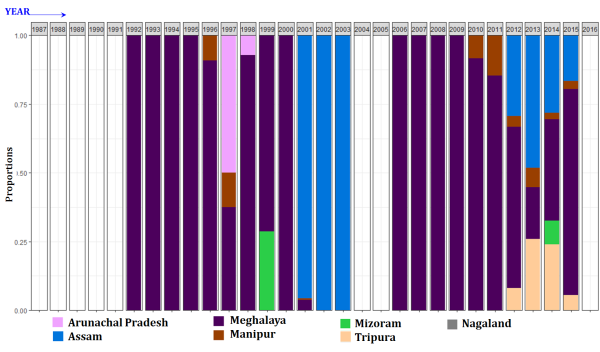


**B**

In North East zone, Meghalaya contributed more number of outbreaks from the year 1992- 2000 and from 2005- 2012 and in year 2015. From year 2001 to 2003 and in year 2012, Assam contributed more than other states. Meghalaya (71.05%) and Assam (19.01%) contributed proportionately more outbreaks compared to other states in the North-East zone (Fig. S1B) and Meghalaya have higher proportion (97.60%) of cattle population.


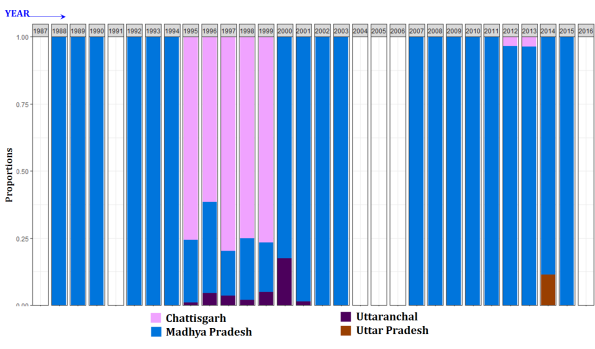


**C**

In Central zone, Madhya Pradesh contributed more number of outbreaks from the years 1988- 1990, 1992-1994, 2000-2003, 2007-2015. From year 1995 to 1999, Chattisgarh contributed more number of outbreaks than other states. Madhya Pradesh (82.43%) and Chhattisgarh (15.64%) contributed maximum number of outbreaks in Central zone compared to other states (Fig. S1C) and Madhya Pradesh has a higher proportion of cattle (70.53%) population compared to buffalo (29.46%).


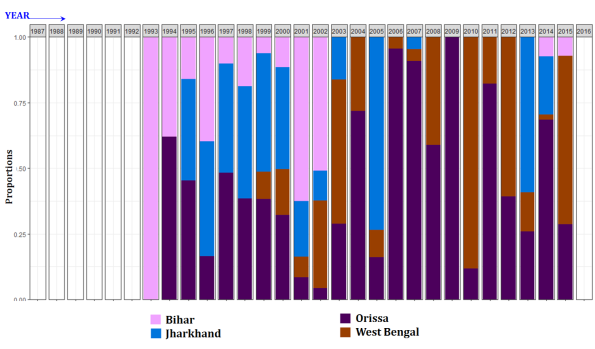


**D**

In East zone, Odisha contributed more number of outbreaks during the years 1994- 1995, 1997, 2004, 2006-2009, 2011 and 2014. However, Bihar contributed to more number of outbreaks in 1993, 2001, 2002. In years 2003, 2010, 2012 and 2015 West Bengal contributed more number of outbreaks compared to other states. In the East zone, West Bengal (44.01%) and Odisha (20.01%) contributed more outbreaks compared to Jharkhand (19.95%) and Bihar (16%) (Fig, S1D) and West Bengal has a higher proportion of cattle (96.51%) compared to buffalo.


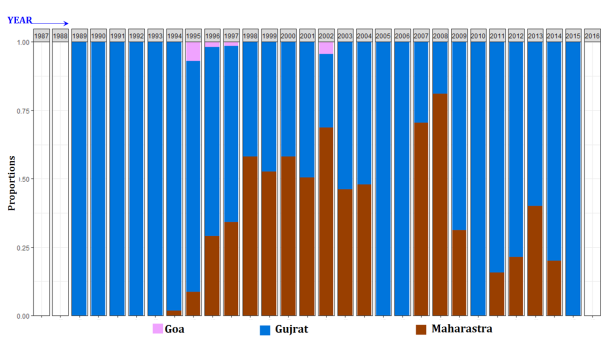


**E**

In West zone, Gujarat contributed more number of outbreaks during the years 1989- 1997, 2003-2005, 2009-2015. However, Maharashtra contributed to more number of outbreaks in years 1998-2002, 2007- 2008. Gujarat (72.21 %) and Maharashtra (27.23%) states had contributed more outbreaks to West zone compared to other states (Fig. S1E) and Gujarat has almost equal proportion of cattle (49.01%) and buffalo (50.98%) population.


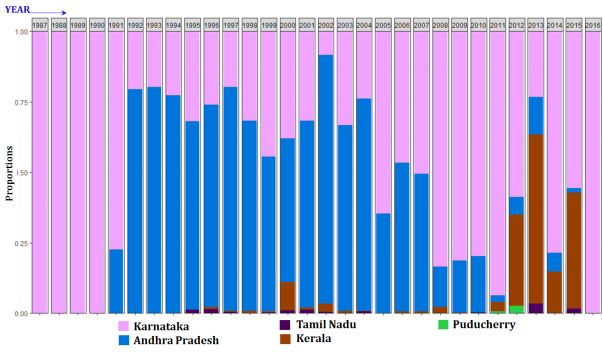


**F**

In South zone, Karnataka contributed more number of outbreaks during the years 1987- 1991, 2005-2012, 2014-2015. Andhra Pradesh contributed more number of HS outbreaks from 1992-2004. Karnataka (54.86%) and Andhra Pradesh (38.91%) contributed more outbreaks to the South zone compared to the rest of the states (Fig. S1F). Karnataka has a higher proportion of cattle (73.27%) compared to buffaloes (26.72%) and Andhra Pradesh has 47.46% of cattle and 52.53% of buffalo population.

**2.** **Proportion of cattle and buffalo in comparison to HS outbreaks**

The proportion of cattle and buffalo and proportion of HS outbreaks in each zone is

shown in Figure S2.

*Figure S2: Proportion of cattle and buffalo in comparison to HS outbreaks for each*

*zone*

The comparison of proportion of the cattle and buffalo population that were recorded in each state (Fig. S3).

*
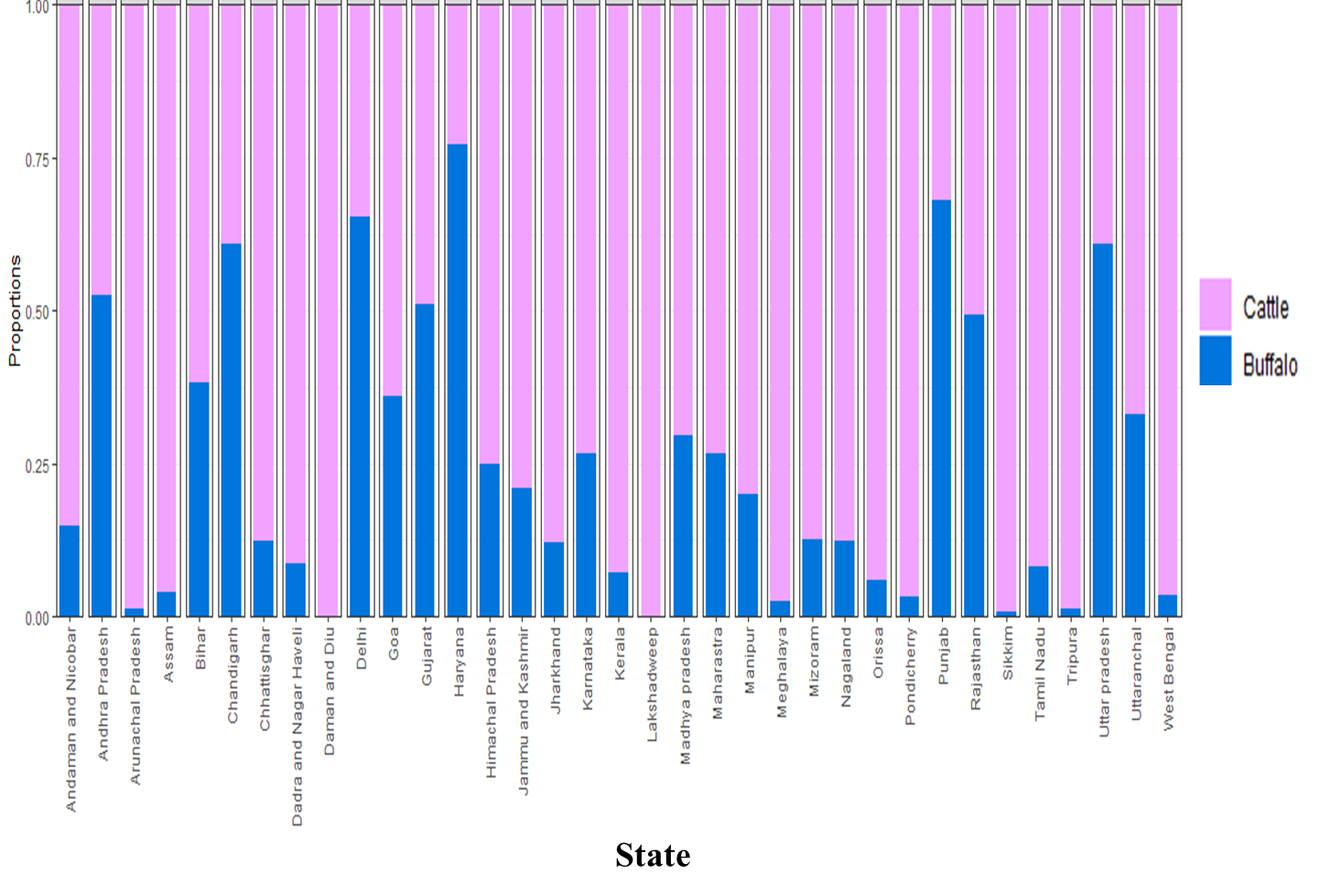
*

*Figure S3: Comparison of Proportion of cattle and buffalo population in different states*

6. Cattle and Buffalo population in different states of India: The cattle (Fig. S4 a ) and Buffalo (Fig. S4 b) population in different states of India


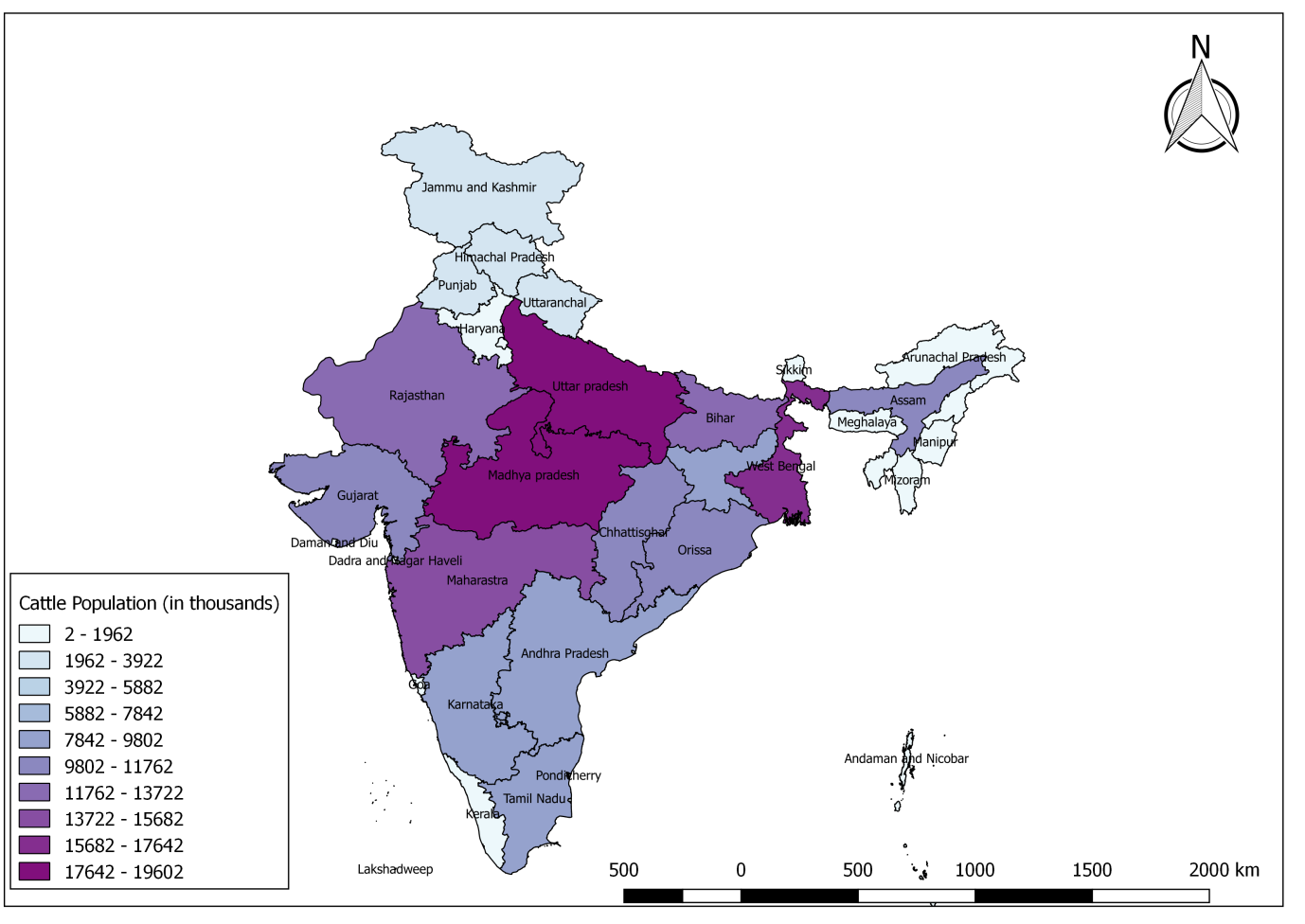


**a**


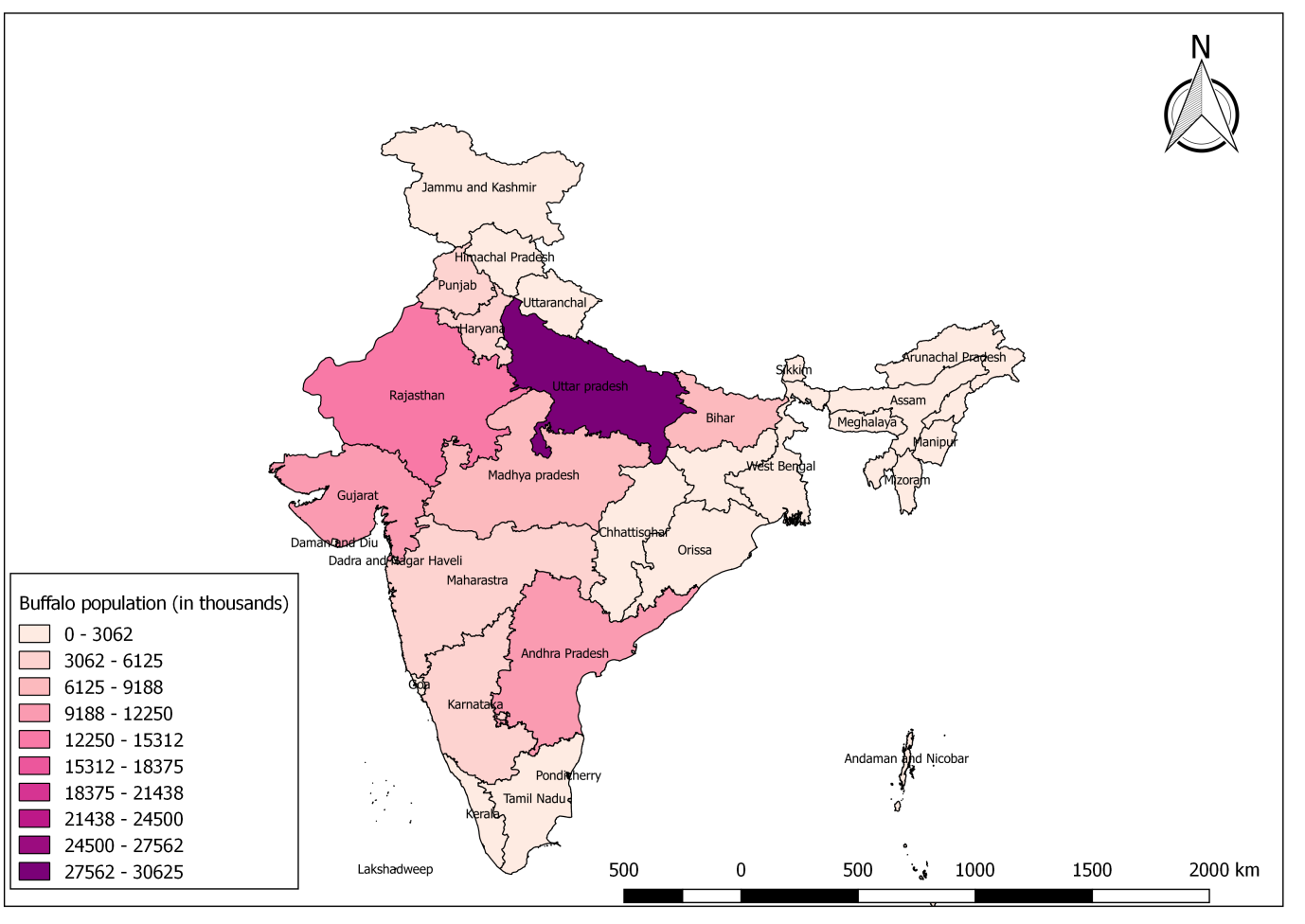


**b**

*Figure S4 : Cattle (a) and Buffalo (b) population in different states of India. The livestock population data was obtained from* [*http://dahd.nic.in/about-us/divisions/statistics*](http://dahd.nic.in/about-us/divisions/statistics) *(Accessed on 7/06/2019). Maps were prepared using QGIS (*[*http://qgis.org*](http://qgis.org/)*)*

**3. Autocorrelation functions (ACF) and partial autocorrelation functions (PACF) for HS outbreaks in different zones:** Autocorrelation functions (ACF) and partial autocorrelation functions (PACF) were calculated to identify whether HS outbreaks were stationary, exhibiting a constant mean and constant variance for all the zones. Resulting plots were examined to identify significant autocorrelations in each variable at multiple lag distances. The ACFs and PACFs analyses were performed using the *tsa* and *forecast* packages in R. Monthly zonal time series was decomposed to describe the monthly trend, seasonality and random noise by additive moving average model.

The autocorrelation function (ACF) and partial autocorrelation function (PACF) plots (Figs. S5A & S6A) of HS outbreaks in North zone shows significant autocorrelation indicated by the areas above the blue dotted horizontal line (95% confidence interval), but the time series appears to be non-stationary. North zone monthly time series shows varying trend in different years (Fig. S7A).

The ACF and PACF plots (Figs. S5B & S6B) of HS outbreaks in North Eastern zone shows significant autocorrelation, but the time series appears to be non-stationary. North Eastern zone monthly time series shows varying trend in different years (Fig. S7B).

The ACF and PACF plots (Figs. S5C & S6C) of HS outbreaks in Central zone shows significant autocorrelation, but the time series appears to be non-stationary. Central zone monthly time series shows decreasing trend from the year 2000 onwards (Fig. S7C).

The ACF and PACF plots (Figs. S5D & S6D) of HS outbreaks in Eastern zone shows significant autocorrelation, but the time series appears to be non-stationary. Eastern zone monthly time series shows varying trend in different years. There is decreasing trend from the year 2005 onwards (Fig. S7D).

The ACF and PACF plots (Figs. S5E & S6E) of HS outbreaks in West zone shows significant autocorrelation, but the time series appears to be non-stationary. Western zone time series shows decreasing trend from the year 2000 onwards (Fig. S7E).

The ACF and PACF plots (Figs. S5F & S6F) of HS outbreaks in South zone shows significant autocorrelation indicated by the areas above the blue dotted horizontal line (95% confidence interval), but the time series appears to be non-stationary. Southern zone time series shows decreasing trend (Fig. S7F).

^
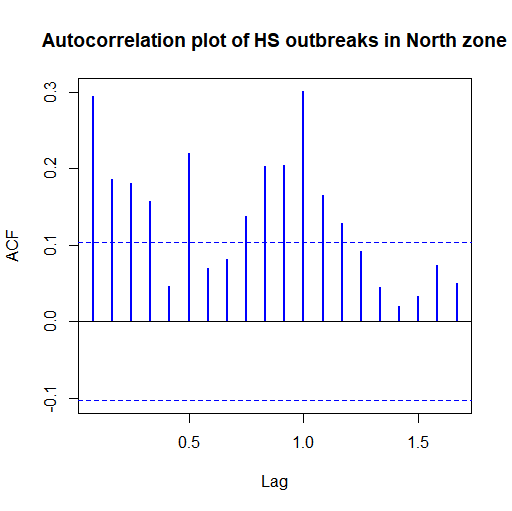

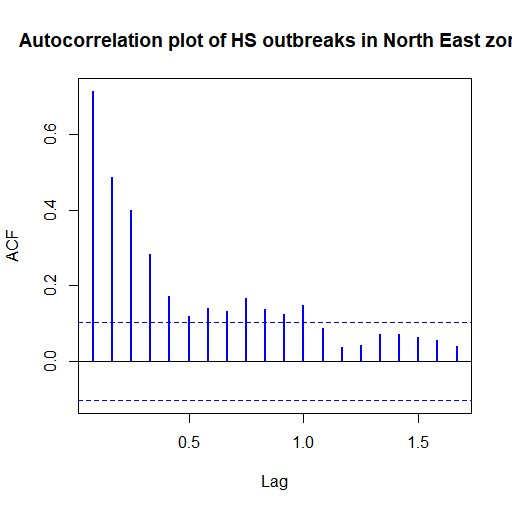
^

**F**

**A**

**B**

**C**

**D**

**E**

^
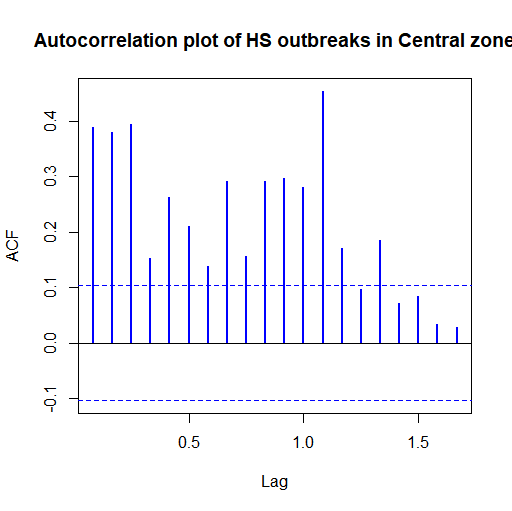

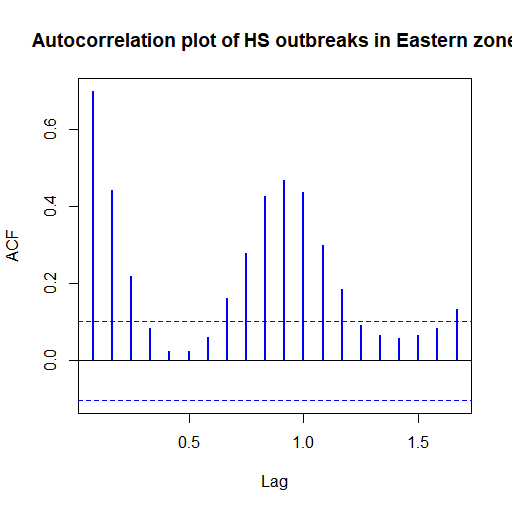
^

^
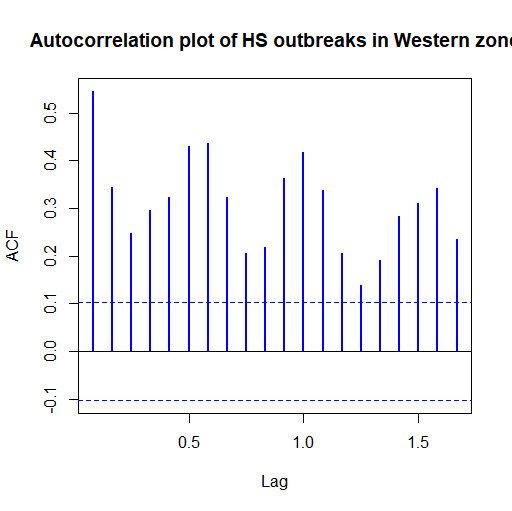

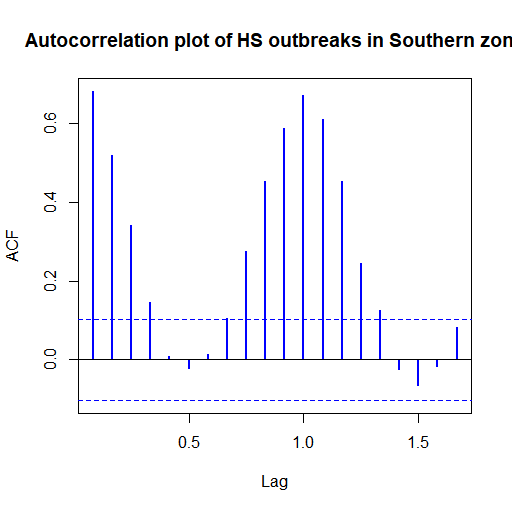
^

*Figure S5: Plots of Autocorrelation function (ACF) HS outbreaks in different zones. The x-axis gives the number of lags in years and the y-axis gives the value of the correlation between -1 and 1. Blue dashed lines indicate the 95% confidence intervals, within which the correlation is non-significant. (A) North zone, (B) North East zone, (C) Central zone, (D) East zone, (E) West zone , (F) South zone.*

^
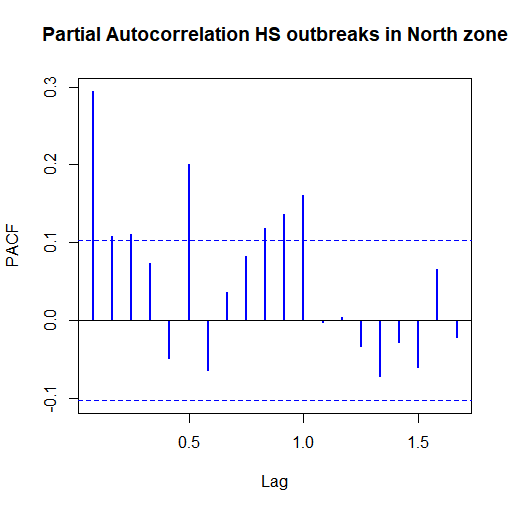

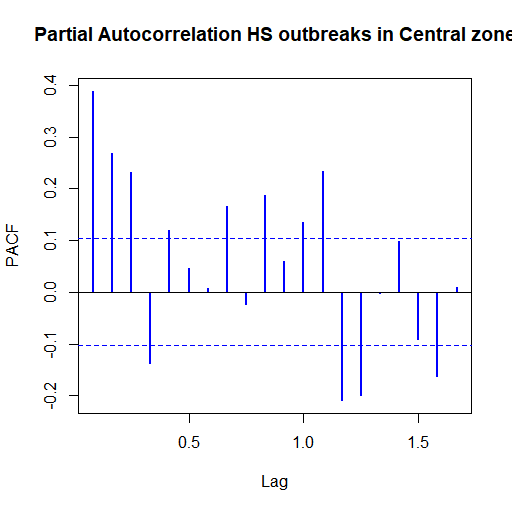
^

**F**

**A**

**B**

**C**

**D**

**E**

^
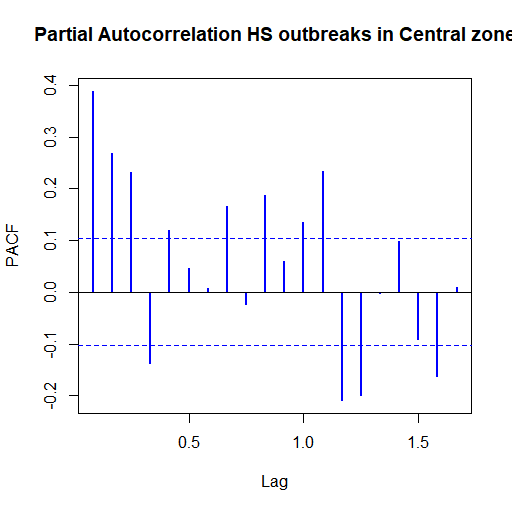

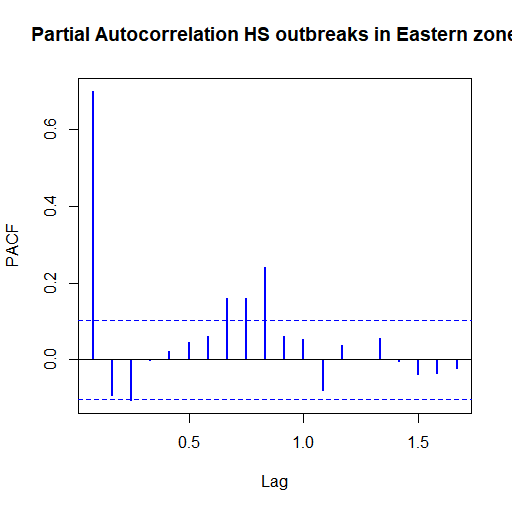
^

^
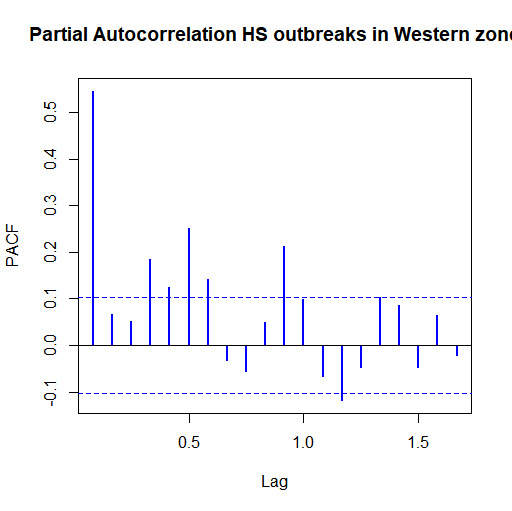

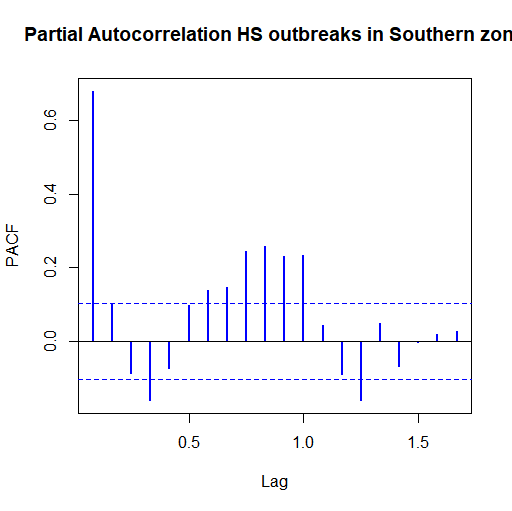
^

*Figure S6: Plots of Partial Autocorrelation function (PACF) HS outbreaks in different zones. The x-axis gives the number of lags in years and the y-axis gives the value of the correlation between -1 and 1. Blue dashed lines indicate the 95% confidence intervals, within which the correlation is non-significant. (A) North zone, (B) North East zone, (C) Central zone, (D) East zone, (E) West zone, (F) South zone.*

^
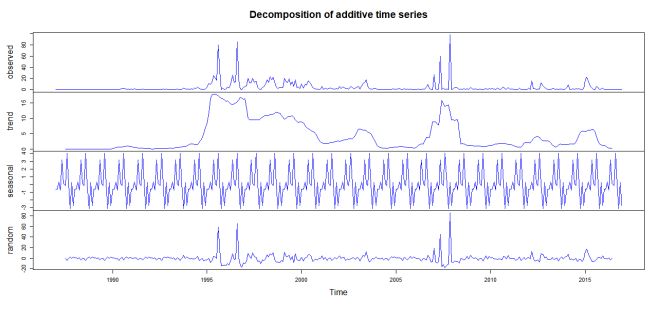

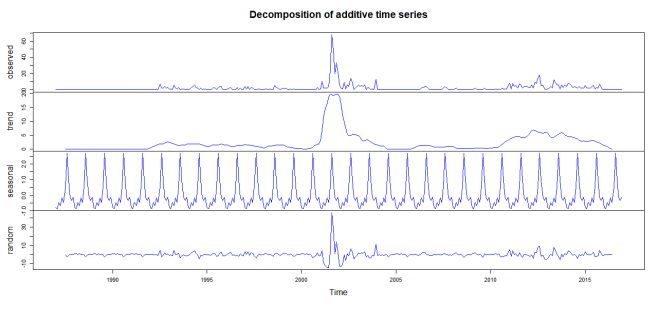
^

**F**

**A**

**B**

**C**

**D**

**E**

^
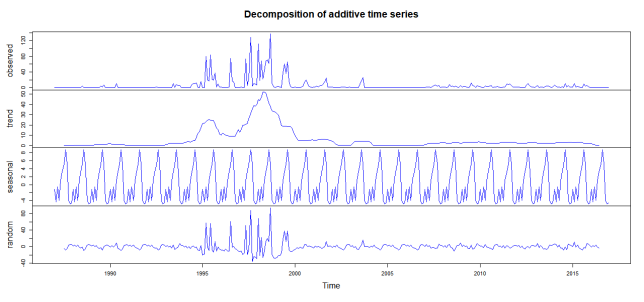

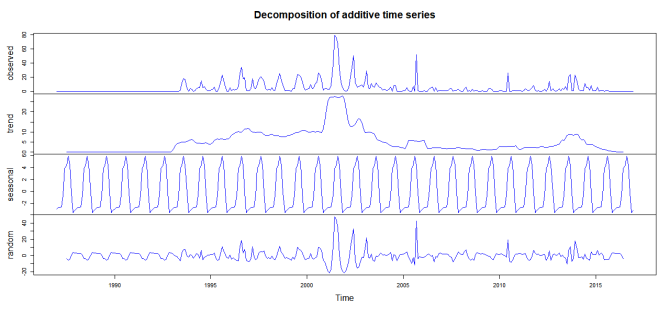
^

^
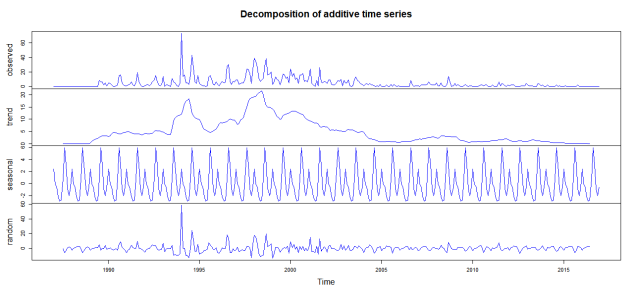

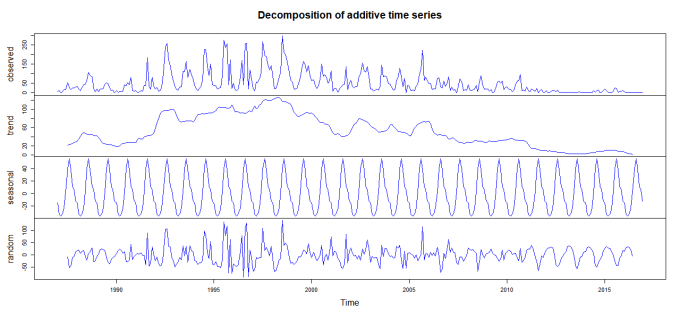
^

*Figure S7: Decomposition of the HS time series into different components: observed, trend, seasonal and random components for different zones (A) North zone, (B) North East zone, (C) Central zone, (D) East zone, (E) West zone and (F) South zone.*

4. Augmented Dickey-Fuller (ADF) and Kwiatkowski-Phillips-Schmidt-Shin (KPSS) tests were performed for all the zonal time series to test whether time series is stationary or not (Table S1). The p-value was significant for all the zonal time series indicating non-stationarity.

| **Zone** | **ADF test (p value)** | **KPSS test (p value)** |
| --- | --- | --- |
| North zone | -4.84450 (0.01) | 0.36875 (0.01) |
| North Eastern zone | -4.7824 (0.01) | 0.11932 (0.09941) |
| Western zone | -3.4508 (0.04758) | 0.68355 (0.01) |
| Southern zone | -5.1597 (0.01) | 0.60522 (0.01) |
| Eastern zone | -4.4109 (0.01) | 0.52103 (0.01) |
| Central zone | -3.8607 (0.01625) | 0.38947 (0.01) |

*Table S1: Augmented Dickey-Fuller and Kwiatkowski-Phillips-Schmidt-Shin tests were performed on the entire zone to test for stationarity.*

**5. Spatial unstructured heterogeneity maps for different zones:** Spatial unstructured heterogeneity maps were plotted to know the importance of distinct district level risk factors responsible for the occurrence of HS outbreaks in different zones (Fig. S8A-F).

**F**

**A**

**B**

**C**

**D**

**E**

^
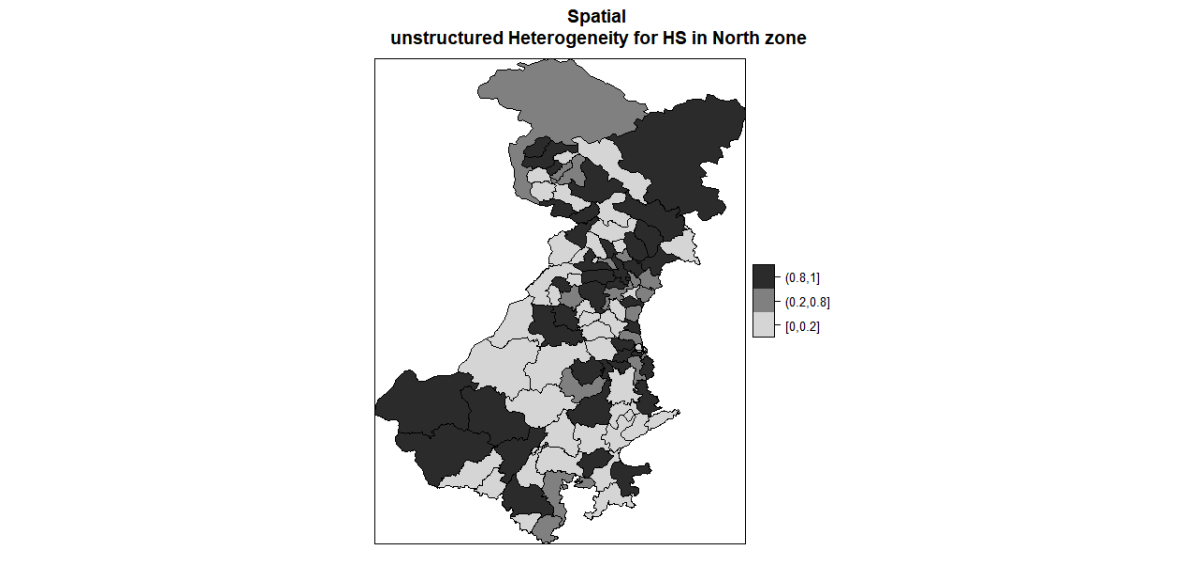
^
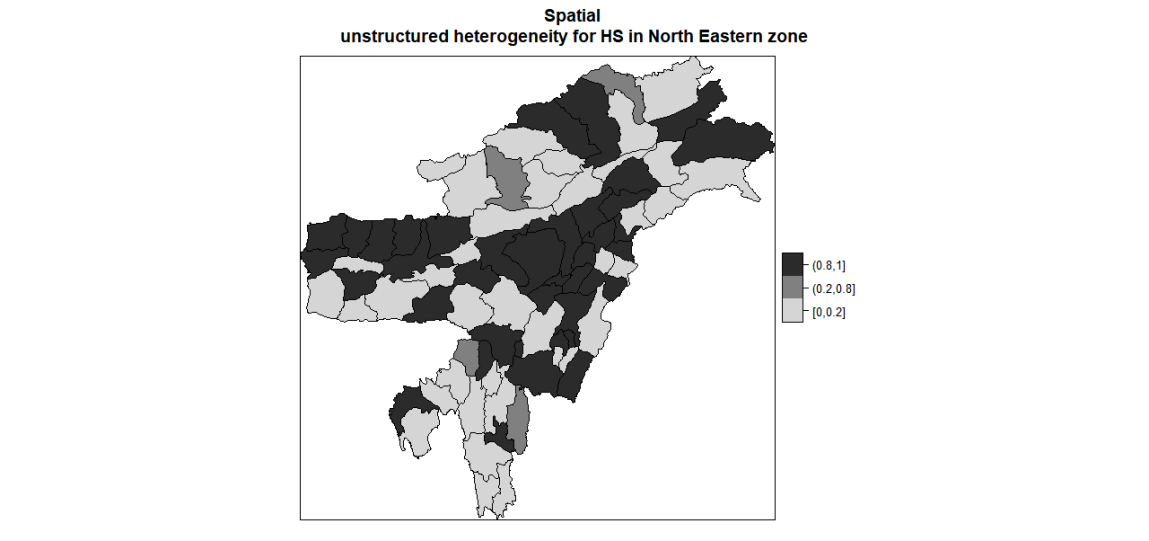


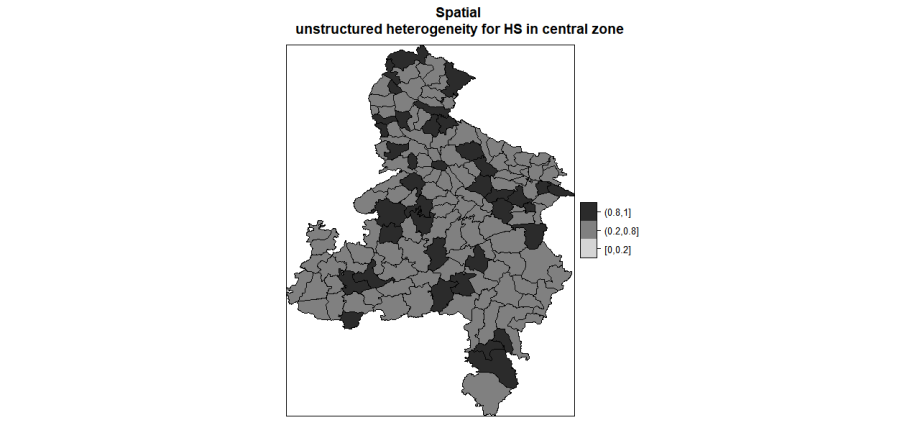

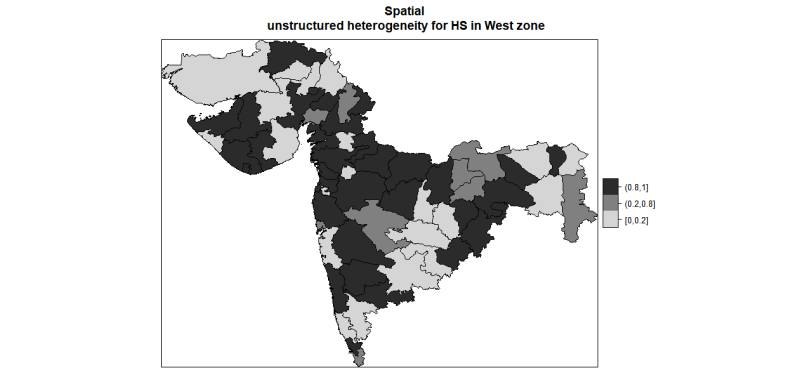


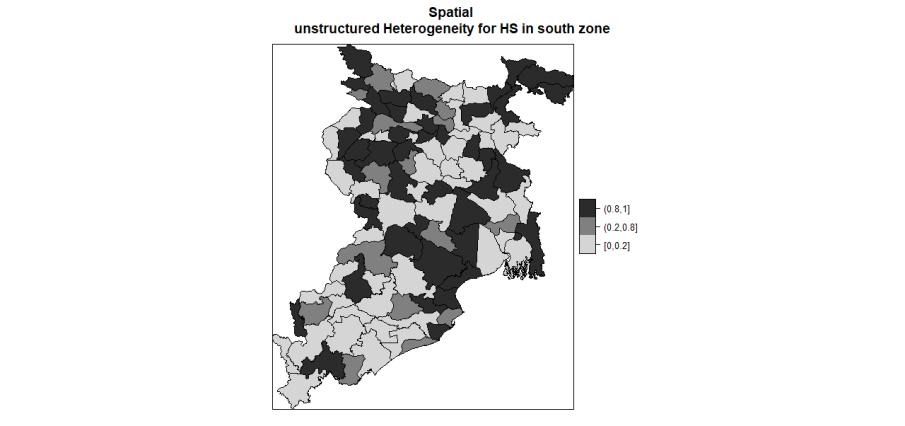

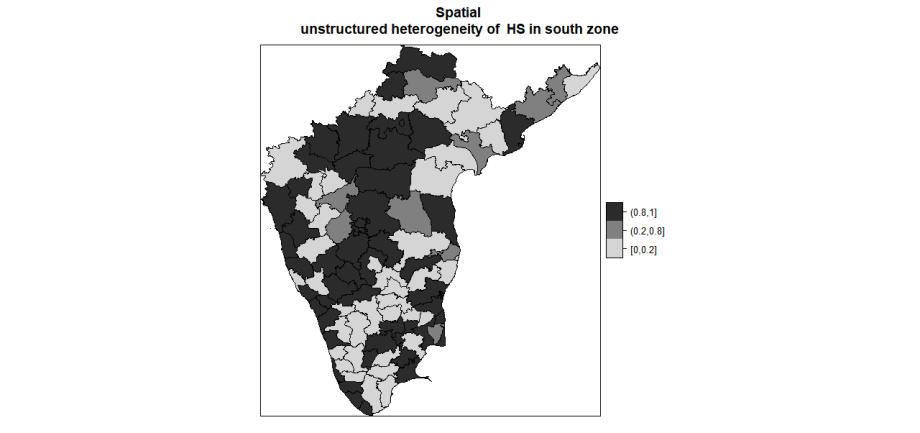


*Figure S8: Spatial unstructured heterogeneity maps for different zones. (A) North zone, (B) North East zone, (C) Central zone, (D) West zone, (E) East zone and (F) South zone. Probabilities are given on a scale of 0-1. Maps output were generated in R.*
